# Boundaries potentiate polycomb response element-mediated silencing

**DOI:** 10.1186/s12915-021-01047-8

**Published:** 2021-06-02

**Authors:** Maksim Erokhin, Fedor Gorbenko, Dmitry Lomaev, Marina Yu Mazina, Anna Mikhailova, Azat K. Garaev, Aleksander Parshikov, Nadezhda E. Vorobyeva, Pavel Georgiev, Paul Schedl, Darya Chetverina

**Affiliations:** 1 Group of Chromatin Biology, Institute of Gene Biology, Russian Academy of Sciences, 34/5 Vavilov St., Moscow, 119334 Russia; 2 Present address: Department of Biology, ETH Zurich, Zurich, Switzerland; 3 Group of Epigenetics, Institute of Gene Biology, Russian Academy of Sciences, 34/5 Vavilov St., Moscow, 119334 Russia; 4 Group of Transcriptional Complexes Dynamics, Institute of Gene Biology, Russian Academy of Sciences, Moscow, Russia; 5 Laboratory of Structural and Functional Organization of Chromosomes, Institute of Gene Biology, Russian Academy of Sciences, 34/5 Vavilov St., Moscow, 119334 Russia; 6 Department of the Control of Genetic Processes, Institute of Gene Biology, Russian Academy of Sciences, 34/5 Vavilov St., Moscow, 119334 Russia; 7 Department of Molecular Biology Princeton University, Princeton, NJ 08544 USA

**Keywords:** Polycomb, Chromatin silencing, Repression, *Bithorax*, *bxd*, *Engrailed*, Architectural protein, Boundary protein, Insulator, Trithorax

## Abstract

**Background:**

Epigenetic memory plays a critical role in the establishment and maintenance of cell identities in multicellular organisms. Polycomb and trithorax group (PcG and TrxG) proteins are responsible for epigenetic memory, and in flies, they are recruited to specialized DNA regulatory elements termed polycomb response elements (PREs). Previous transgene studies have shown that PREs can silence reporter genes outside of their normal context, often by pairing sensitive (PSS) mechanism; however, their silencing activity is non-autonomous and depends upon the surrounding chromatin context. It is not known why PRE activity depends on the local environment or what outside factors can induce silencing.

**Results:**

Using an attP system in *Drosophila*, we find that the so-called neutral chromatin environments vary substantially in their ability to support the silencing activity of the well-characterized *bxd*PRE. In refractory chromosomal contexts, factors required for PcG-silencing are unable to gain access to the PRE. Silencing activity can be rescued by linking the *bxd*PRE to a boundary element (insulator). When placed next to the PRE, the boundaries induce an alteration in chromatin structure enabling factors critical for PcG silencing to gain access to the *bxd*PRE. When placed at a distance from the *bxd*PRE, boundaries induce PSS by bringing the *bxd*PREs on each homolog in close proximity.

**Conclusion:**

This proof-of-concept study demonstrates that the repressing activity of PREs can be induced or enhanced by nearby boundary elements.

**Graphical abstract:**

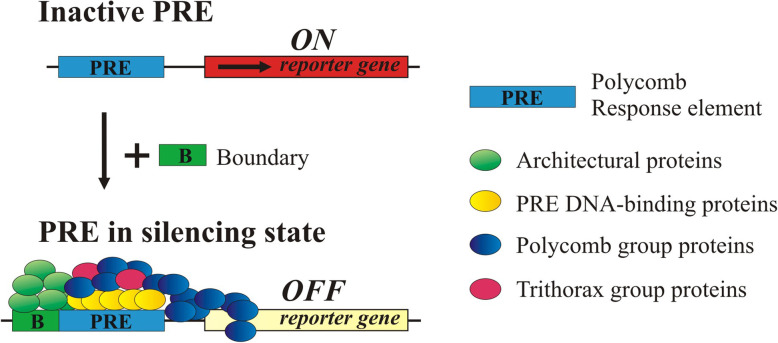

**Supplementary Information:**

The online version contains supplementary material available at 10.1186/s12915-021-01047-8.

## Background

In many developmental systems, the mechanisms involved in the choice of cell fate are distinct from those deployed for maintaining or remembering cell fate decisions. One classic example of choice is the establishment of parasegment identity in the posterior 2/3rds of *Drosophila* embryo by the three bithorax complex (BX-C) homeotic genes: *Ultrabithorax* (*Ubx*), *abdominal-A* (*abd-A*), and *Abdominal-B* (*Abd-B*) [1–3]. These genes are controlled by nine parasegment-specific regulatory domains, which are sequentially activated along the anterior-posterior axis by the combined action of gap, pair-rule, and maternal gene products in blastoderm stage embryos. *Ubx* determines segment identity in two parasegments, PS5 and PS6, and its expression in these parasegments is controlled by the *abx/bx* and *bxd/pbx* regulatory domains, respectively. *abx/bx* is activated at the blastoderm stage in PS5, while *bxd/pbx* is silenced. In PS6, both domains are active, but *Ubx* expression is controlled by *bxd/pbx*.

The maternal and early zygotic gene products that initially determine the appropriate activity state of the BX-C regulatory domains for each parasegment disappear after the onset of gastrulation. Consequently, a different mechanism is required to remember whether the domain is *on* or *off* during the rest of development. The memory mechanism is epigenetic. The *off* or silenced state is remembered by Polycomb group proteins (PcG), while the *on* or active state is maintained by trithorax group proteins (TrxG) [4–10]. PcG and TrxG proteins not only control the transcriptional activity of the Hox complex genes, but also many other genes implicated in different aspects of development and differentiation. Moreover, disruption of the PcG or TrxG maintenance systems can result in developmental abnormalities and other pathologies [11–16].

In *Drosophila*, most of the known PcG proteins are components of three complexes: polycomb repressive complexes 1 and 2 (PRC1 and PRC2) and Pho repressive complex (PhoRC). The canonical PRC1 core subunits are sex combs extra (Sce, also known as dRing), posterior sex combs (Psc), polycomb (Pc), and polyhomeotic (Ph) proteins [17–19]. The PRC2 core subcomplex contains enhancer of zeste (E(z)), extra sex combs (Esc), suppressor of zeste 12 (Su(z)12), and chromatin assembly factor 1 (Caf1) subunits [20, 21]. Finally PhoRC is a DNA-binding complex consisting of Sfmbt [22] and DNA-binding protein pleiohomeotic (Pho) [23]. The TrxG proteins are a more heterogeneous group of proteins which includes the histone H3K4-specific methyltransferase trithorax (Trx), the histone acetyltransferase CBP, and components of chromatin remodeling BAP/PBAP complexes [5, 7, 9, 24, 25].

The PcG and TrxG proteins are recruited to their regulatory targets by specialized DNA elements called PREs (polycomb response elements) [26–29]. PREs are present in each of the BX-C regulatory domains including the two domains, *abx/bx* and *bxd/pbx*, that control *Ubx* expression [30, 31]. They are also found in the chromatin domains associated with other key developmental transcription factors such as *engrailed* and *even-skipped* [32, 33]*.* The binding of PcG/TrxG proteins to PREs is mediated by sequence-specific DNA-binding proteins, including Pho [23, 34] and Combgap [35].

One characteristic activity of PREs that can be readily assayed is their ability to induce pairing-sensitive silencing (PSS) [33, 36]. This phenomenon was first observed when the eye colors of flies carrying transgenes with a *white* reporter and a PRE were compared in hemizygotes and homozygotes. In classical PSS, there is little or no evidence of silencing in hemizygous flies, while strong silencing is evident when the transgene is homozygous. Since PSS is typically observed for transgenes inserted at the same chromosomal site, but not at different sites, the enhancement of repression in homozygotes is thought to be due to *trans* interactions between the two copies of the PRE, one on each homolog [33, 36]. The effects of pairing have also been observed for PREs in their endogenous context [37].

The activity of PRE elements in these transgene experiments is influenced by the surrounding chromatin environment. Depending on the integration site, PREs can repress and have a neutral or even a positive impact on gene transcription [33, 38–46]. While these findings indicate that PRE activity is modulated by the chromosomal context, it is not clear what features of the immediate neighborhood are important and why. Since PREs do not seem to be functionally autonomous, one idea is that other nearby regulatory elements can impact their activities. In this respect, it is interesting to note that in the *Abd-B* region of BX-C, the PREs for the *iab-5*, *iab-6*, *iab-7*, and *iab-8* regulatory domains are located in relatively close proximity to the chromatin boundary elements (insulators) that flank these domains [1, 47–49]. In BX-C, boundary elements define the centromere proximal ends of the regulatory domain: *Mcp* for *iab-5*; *Fab-6* for *iab-6*; *Fab-7* for *iab-7*, and *Fab-8* for *iab-8* [1]. Moreover, at least in the case of *Fab-7* and *Mcp*, the PRE and boundary are integral parts of the same regulatory element [37, 50–52]. The close linkage between chromatin boundaries and PREs raises the possibility that these architectural elements might facilitate the acquisition of silencing activity by PREs. Consistent with this idea, self-pairing interactions between the Su(Hw) and the *Mcp* boundaries were shown to facilitate PSS between transgene inserts megabases from each other and even between transgenes inserted on different chromosomes [53–56]. Similar results have been obtained in transgene assays [57].

In the present study, we tested whether a close linkage between boundaries and PREs can impact the acquisition of silencing activity. We show that in chromosomal contexts that are refractory to PRE-induced silencing, the PREs fail to recruit PcG/TrxG proteins. However, both silencing and recruitment of PRE-associated proteins can be triggered by linking the PRE to an artificial boundary element consisting of multimerized binding sites for the Su(Hw), CTCF, or Pita polydactyl zinc finger DNA-binding proteins. Two different mechanisms, one that occurs in *cis* and the other in *trans* appear to be in play. The first is observed when the boundary is placed in close proximity to the PRE. In this case, silencing is activated in fly hemizygous for the transgene insert and is enhanced when the transgene is homozygous. The induction of silencing activity in hemizygotes is not dependent on the identity of the linked boundary and is observed for all three boundaries. ChIP experiments indicate that the linked boundary facilitates alterations in the local chromatin structure. In the region spanning the PRE, there is a depletion of histone H3 levels and a concomitant recruitment of factors required for PRE function. Interestingly, in the region to either side of the boundary, PRE sequences histone H3 levels are increased. The second mechanism is observed when the boundary element is separated by 1 kb or more from the PRE. In this case, silencing is not or at most only weakly activated in hemizygotes. However, silencing activity is induced in homozygous flies. In this case, the mechanism is dependent upon boundary:boundary pairing interactions as it is not observed when the PREs are linked to heterologous instead of homologous boundaries.

## Results

### *bxd*PRE silencing activity depends on the chromosomal context

**Fig. 1:**
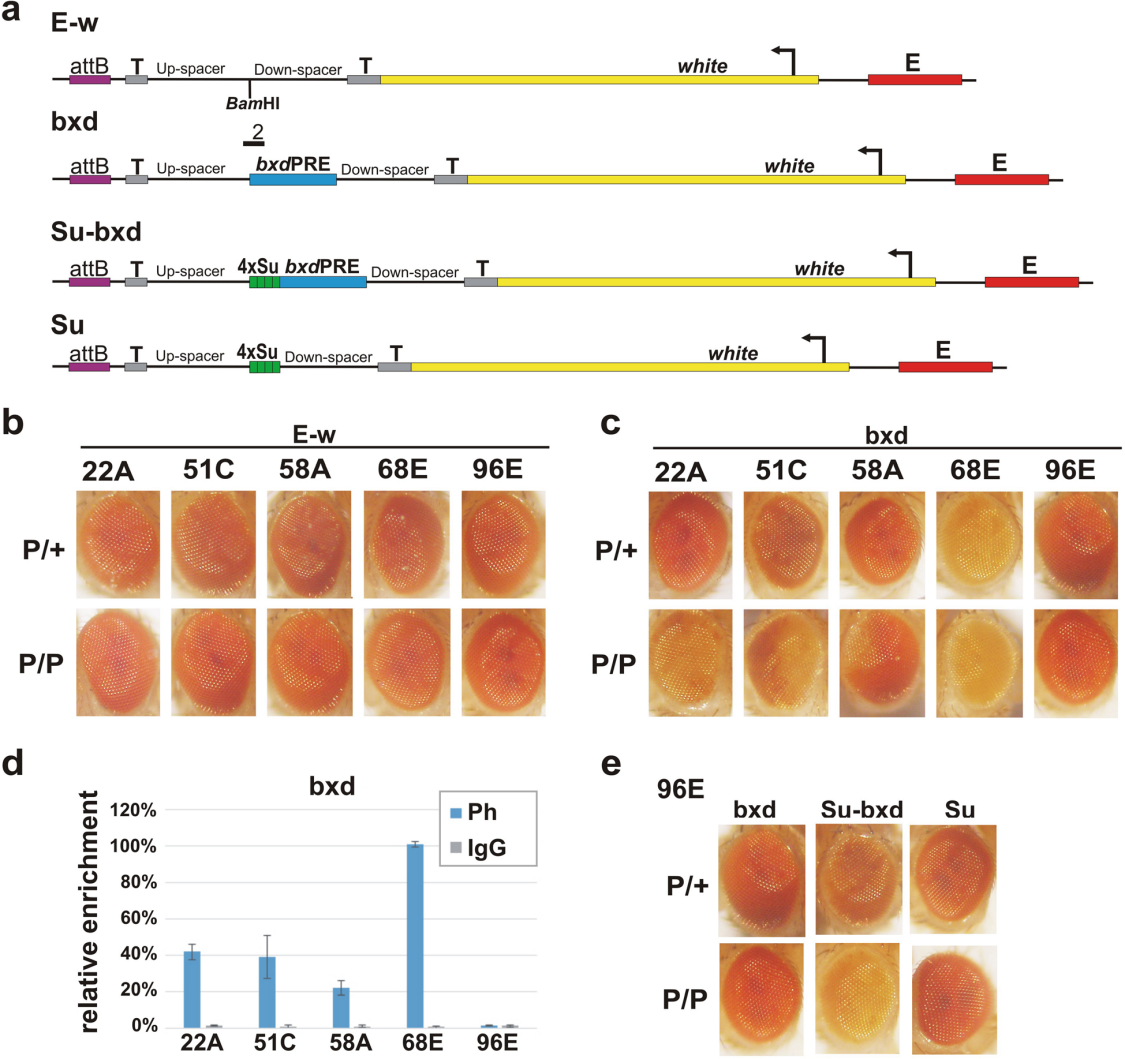
Su(Hw) binding sites induce *bxd*PRE silencing activity. **a** Map of transgenes. Labels: “attB” – attB site required for transgene integration into the attP insertion site; “*bxd*PRE” – *bxd*PRE silencing element; “T,” terminators of transcription; *white* -marker gene; “E” - enhancer of the *white* gene, “4xSu” – four binding sites for Su(Hw) protein. **b** The eye phenotypes of flies that have the control “E-w” transgene integrated into the different attP sites: 22A (Bloomington Drosophila Stock Center (BDSC) #24481), 51C (BDSC #24482), 58A (BDSC #24484), 68E (BDSC #24485), 96E (BDSC #24487). P/+, hemizygous; P/P, homozygous adult flies. **c** The eye phenotypes of flies that have the “bxd” transgene integrated into the different attP sites: 22A, 51C, 58A, 68E, and 96E. **d** Ph enrichment at *bxd*PRE in “bxd” transgenes integrated into the different attP insertion sites. Diagrams summarize the results of X-ChIP with Ph antibody or with IgG from a non-immunized animal as a negative control. X-ChIP was analyzed by real-time PCR with primers specific to transgene *bxd*PRE—the region indicated by underlined number 2 above the “bxd” transgene map. The X-ChIP experiments were performed with chromatin isolated from the heads of homozygous adult flies. The ordinate shows the percentage of target sequences in the immunoprecipitated material relative to the input DNA and normalized to the positive control—a sequence adjacent to the endogenous *bxd*PRE in BX-C (*bxd*PRE-Genome). The line with bxd transgene is designated by its attP insertion site and is indicated on the abscissa. Vertical lines indicate SDs. **e** The eye phenotypes of flies with the “bxd,” “Su-bxd,” or “Su” transgenes at the 96E attP site

In previous studies, PREs were found to repress expression of reporter genes in only about half of the transgene insertion sites [38–41, 58, 59]. To better understand the context-dependent factors that impact PRE activity, we used the ΦC31 site-specific integration system [60] to generate independent *Drosophila* transgenic lines. For this purpose, we selected five attP sites that had previously been shown to provide a context in which the expression of a *white* reporter is not subject to obvious repression or activation by the surrounding chromatin neighborhood (Additional file 1: Table S1). Three attP sites are on the 2nd chromosome (22A, 51C, and 58A), while two are on the 3rd chromosome (68E and 96E). As a PRE, we selected a well-characterized 656-bp *bxd*PRE element from the *Ubx* regulatory region [30, 31, 61, 62] (bxd construct, Fig. 1a). To evaluate the silencing activity of the *bxd*PRE as a reporter, we used a *white* gene with its eye tissue-specific enhancer (E). The *bxd*PRE was flanked by ~1 kb “neutral” spacers derived from coding regions of the eGFP and RFP genes and by terminators of transcription (SV40 terminator upstream and yellow gene terminator downstream) to reduce the influence of potential transcription from surrounding genomic sequences. To better assess the effects of the PRE, we also inserted a control construct at each site, which has all of these components except for *bxd*PRE (E-w construct, Fig. 1a). The transgene constructs were inserted in *w*^-^ attP lines lacking a functional *white* gene. The silencing activity of the *bxd*PRE was assessed by the reduction in eye pigmentation as pigmentation is known to be directly correlated with the level of *white* gene transcription [62, 63].

Shown in Fig. 1b, c are the eye color phenotypes of hemizygous and homozygous flies for each of the five insertion sites. In the five lines carrying the E-w control transgene, there is no evidence of repression in either hemizygotes or homozygotes (Fig. 1b). In contrast, different degrees of repression are observed in 4 of the 5 lines with the bxd transgene (Fig. 1c). Line 22A is a classic example of PSS. It shows little or no evidence of silencing as a hemizygote, while as a homozygote there is a strong and almost uniform reduction in pigmentation. Though PSS is observed for line 58A, it differs from line 22A in that only a small sector of the eye shows a significant loss of pigmentation as a homozygote. Unlike 22A and 58A, weak silencing is observed in line 51С as a hemizygote. However, PSS is also evident as silencing is clearly enhanced when the flies are homozygous for the insert. For line 68E, eye pigmentation is greatly reduced in both hemizygous and homozygous flies. Finally, the *bxd*PRE is unable to induce silencing at 96E insertion site, suggesting that this chromatin environment renders the PRE inactive. In all cases, flies within a given line have similar eye pigmentation phenotypes. Since PSS is thought to be dependent upon homolog pairing, the different properties of the five insertion sites could be due to differences in the strength of local homolog pairing. To investigate this possibility, we took advantage of a recent genome-wide study that measured interaction frequencies between homologs at the embryo stage [64]. However, analysis of the available Hi-C data did not show any significant correlations (Additional file 2).

Silencing is expected to be accompanied by the association of PcG proteins with the *bxd*PRE. To confirm that this is the case, we isolated chromatin from adult heads of homozygous *bxd*PRE transgene flies and performed chromatin immunoprecipitation (X-ChIP) with antibodies against Ph, which is a core component of the PRC1 complex (Fig. 1d). To compare Ph association in different lines, we calculated the extent of enrichment relative to an internal positive control. For this purpose, we used primers to a sequence immediately adjacent to the endogenous *bxd*PRE (hereafter referred to as *bxd*PRE-Genome) that is known to be enriched in PcG/TrxG proteins. Figure 1c shows that Ph is associated with the transgenic *bxd*PRE in the four lines that show silencing of the *white* gene. Moreover, the extent of association correlates well with the level of repression observed in each line. Consistent with the lack of silencing of *white* in the 96E insertion, Ph is not found to be associated with its *bxd*PRE sequence.

### Multimerized sites for Su(Hw) boundary induce *bxd*PRE silencing at 96E

PREs are often located near other transcriptional regulatory elements. As noted above, the PREs in the four *Abd-B* regulatory domains are positioned close to the boundary elements for each domain. Another example is one of the PREs for the *even-skipped* (*eve*) gene that is located next to the distal boundary of the *eve* locus *homie* [32]. These observations led us to wonder whether boundary elements might be able to augment the silencing activities of PREs.

To explore this possibility, we selected the 96E attP since the *bxd*PRE is unable to silence *white* at this insertion site. As the test boundary, we used an artificial element consisting of multimerized binding sites for the polydactyl zinc finger protein Su(Hw) rather than an endogenous boundary. Endogenous boundaries contain binding sites for many different proteins, and some are known to be required for PRE activity. For example, the GAF protein is implicated not only in insulation but also in PcG-dependent silencing [65].

The Su(Hw) protein is responsible for the boundary activity of the insulator element associated with the *gypsy* transposon [66, 67]. The *gypsy* transposon has 12 binding sites for the Su(Hw) protein [68, 69]; however, previous studies have shown that a multimer consisting of only four copies of the third Su(Hw) binding site from the *gypsy* insulator is sufficient for boundary activity in transgene reporter assays [70] and in the context of BX-C [71]. This 4xSu(Hw) multimer was placed on the distal side of the *bxd*PRE (Fig. 1a: Su-bxd construct) so that the PRE is between it and the *white* gene. In this position, the 4xSu(Hw) multimer would not be able to insulate *white* from PRE-dependent silencing [54, 57, 72]. To assess the effects of the multimer alone, we inserted a control transgene containing 4xSu(Hw) but not the PRE (Fig. 1a: Su construct). Figure 1e shows that combining 4xSu(Hw) with the *bxd*PRE has a dramatic effect on *white* expression. Silencing of *white* is evident in hemizygotes, while strong PSS is observed when the transgene is homozygous. In contrast, the 4xSu(Hw) multimer alone has no effect on eye pigmentation either as a hemizygote or a homozygote. Thus, the presence of the 4xSu(Hw) multimer can induce the establishment of silencing by the *bxd*PRE in a chromosomal location that is not conducive to PcG-dependent silencing.

### Su(Hw) binding facilitates recruitment of PcG/TrxG and PRE DNA-binding proteins to the *bxd*PRE at 96E

**Fig. 2:**
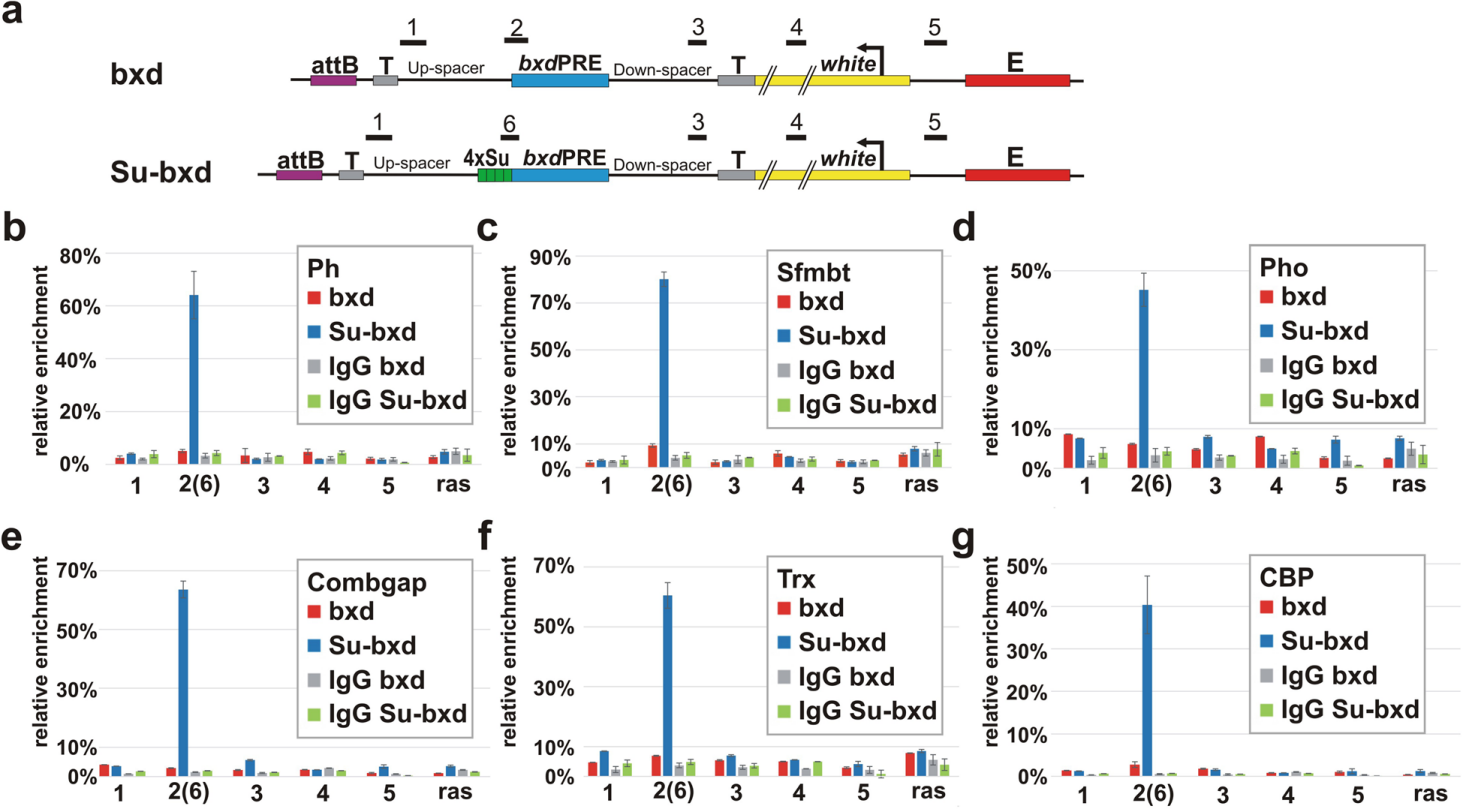
Su(Hw) boundary induces the recruitment of PcG proteins to the *bxd*PRE. (**a**) Maps of the “bxd” and “Su-bxd” transgene constructs. The numbers above the maps (1, 2, 3, 4, 5, 6) indicate the regions amplified by qPCR in X-ChIP experiments. The X- ChIPs were performed with chromatin isolated from heads of adult flies homozygous for the “bxd” or “Su-bxd” transgenes at 96E insertion site. The X-ChIPs were performed with specific antibodies or with IgG. The specific antibodies: (**b**) Ph, (**c**) Sfmbt, (**d**) Pho, (**e**) Combgap, (**f**) Trx, (**g**) CBP. The ordinate shows the percentage of target sequences in the immunoprecipitated material relative to the input DNA and normalized to *bxd*PRE-Genome. The transgene specific regions and negative genome control (ras - coding part of Ras64B gene) are indicated on the abscissa. Other designations are as in Fig. 1

As shown above, Ph is recruited to *bxd*PRE insertions that are able to repress *white* expression but is not found associated with the *bxd*PRE at 96E. If the addition of the 4xSu(Hw) multimer induces PcG-dependent repression, it should also facilitate the recruitment of Ph to the 96E *bxd*PRE. To test this prediction, we used ChIP to examine Ph association at 5 sites in the Su-bxd and bxd transgenes: (1) the distal end of the spacer sequence upstream of *bxd*PRE, (2) *bxd*PRE, (3) the distal end of the spacer sequence downstream of *bxd*PRE, (4) the *white* transcription unit, and (5) the *white* promoter (Fig. 2a). As a negative control, we used the Ras64B coding region, while the bxdPRE-*Genome* region was used as a positive internal control.

As would be predicted from the activation of silencing, there is a substantial increase in Ph association with the *bxd*PRE in the Su-bxd transgene as compared to the bxd transgene (Fig. 2b). While Ph is enriched at the *bxd*PRE, its association with the four other sites in the Su-bxd transgene is essentially the same as in the bxd transgene or the negative Ras64B control. This result is consistent with previous ChIP experiments in which we found that Ph (as well as several other PcG proteins: see below) associates with the *bxd*PRE element in the transgene construct, but not with other sequences even though *white* expression is silenced [62]. Like silencing, the recruitment of Ph requires a combination of the *bxd*PRE and the 4xSu(Hw) multimer as Ph is not associated with the control transgene carrying only the 4xSu(Hw) multimer (Additional file 1: Figure S1b). Consistent with the idea that the presence of Su(Hw) protein bound to its target sites is responsible for the acquisition of silencing activity and the recruitment of Ph, we find that Su(Hw) associates with the 4x multimer not only in the Su transgene but also the Su-bxd transgene (Additional file 1: Figure S1c).

Biochemical and genetic studies have shown that, like other PREs, the silencing activity of the *bxd*PRE depends upon several DNA-binding proteins that help recruit the PRC1 and PRC2 complexes. Thus, one explanation for the inability of the *bxd*PRE element alone to silence *white* at 96E is that these DNA-binding proteins are unable to associate with the *bxd*PRE. In this model, these proteins would be able to access their recognition sequences in the PRE when the 4xSu(Hw) multimer is included in the transgene, but not when it is absent. The DNA-binding proteins known or thought to be important for *bxd*PRE silencing include Pho, which binds PREs together with its partner Sfmbt (the PhoRC complex) as well as the Combgap DNA-binding protein. An alternative model is that the *bxd*PRE at 96E is unable to silence because it is occupied by TrxG proteins and these factors block association of Ph and other PcG proteins and/or their function. In this case, the presence of the 4xSu(Hw) multimer could shift the balance to favor of the recruitment of PcG complexes.

To test these two models, we used antibodies against Pho, Sfmbt, Combgap, and two TrxG proteins, Trx and CBP, for ChIP experiments. In the bxd transgene, we observe only background levels of Pho, Sfmbt, and Combgap in ChIPs for the PRE and other sequences in the transgene (Fig. 2c–e). In contrast, all three of these proteins are detected at the *bxd*PRE in the Su-bxd transgene. These results are consistent with the predictions of the first model. We infer from these finding that the presence of the 4xSu(Hw) multimer induces the association of key DNA-binding proteins with the *bxd*PRE.

The second model predicts that TrxG proteins will be associated with the transgene containing the *bxd*PRE alone, but will be displaced by PcG proteins when the 4xSu(Hw) multimer is present. However, like the PcG proteins, the Trx and CBP are recruited to the *bxd*PRE only when the 4xSu(Hw) multimer is present (Fig. 2f, g). Thus, the boundary induces the association of PRE DNA-binding proteins as well as both PcG and TrxG factors to the *bxd*PRE. It remains to be determined whether this is actual co-occupancy or whether it reflects instead a heterogeneity in the population such that some PREs are occupied by PcG proteins while others are occupied by TrxG proteins.

### The Su(Hw) multimer augments *bxd*PRE silencing activity at different chromosomal sites

**Fig. 3:**
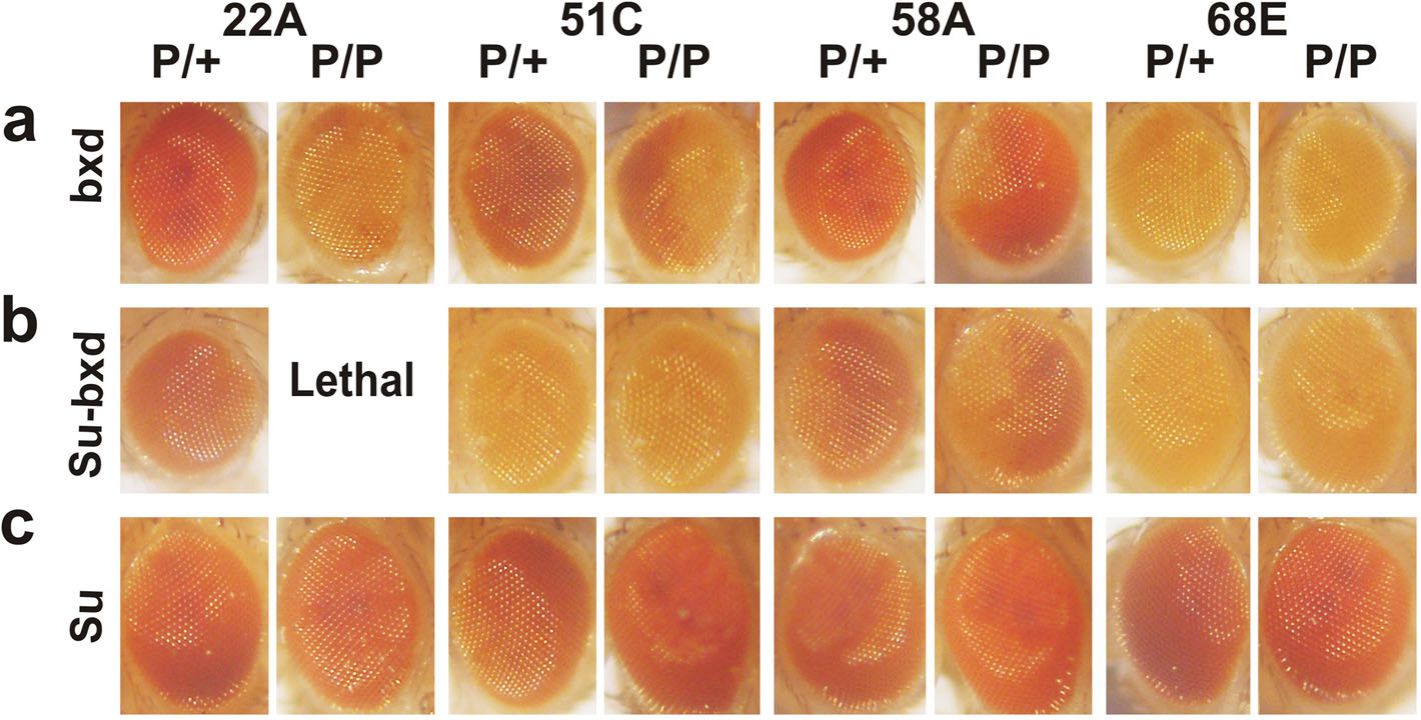
Su(Hw) multimer can stimulate *bxd*PRE silencing activity at different insertion sites. 22A, 51C, 58A and 68E – designates the chromosome position of attP insertion sites. Phenotypes of eyes of adult flies with (**a**) bxd, (**b**) Su-bxd, and (**c**) Su transgenes in hemizygotes (P/+) and homozygotes (P/P)

**Fig. 4:**
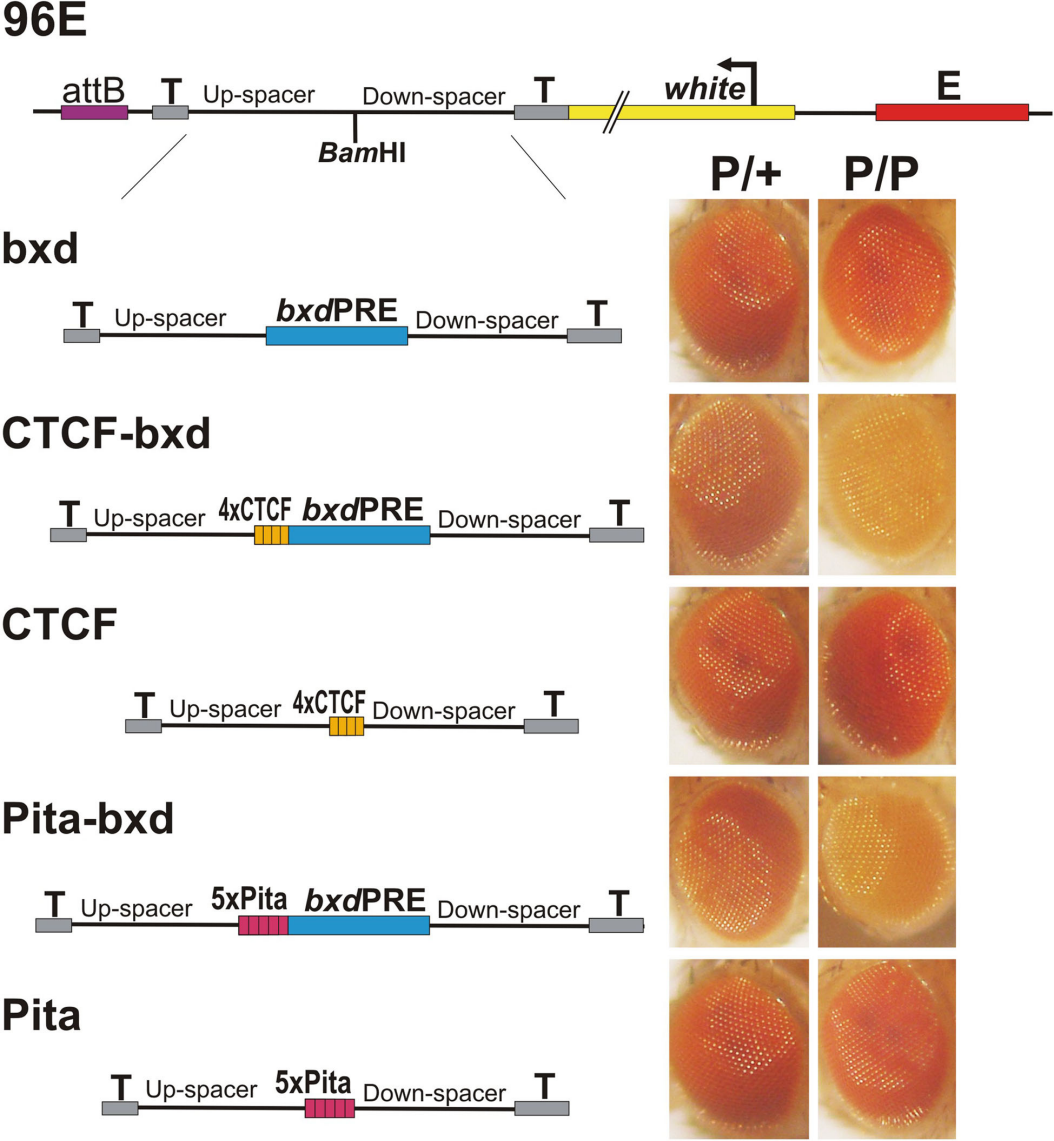
CTCF and Pita multimers induce *bxd*PRE silencing activity. Transgenes were integrated into 96E attP insertion site. Designations: “4xCTCF”––four binding sites for CTCF protein; “5xPita”—five binding sites for Pita protein. Other designations are as on Fig. 1

Genome-wide ChIPs indicate there is a minor Su(Hw) peak that overlaps the attP site at 96E, while there are two larger peaks on either side of the attP ~5 kb and ~10 kb away (Additional file 3, data from [73, 74]). This raises the possibility that the 4xSu(Hw) multimer is able to rescue the silencing activity of the *bxd*PRE at this particular attP site only because of the endogenous Su(Hw) protein. For this reason, we asked whether the 4xSu(Hw) multimer is able to augment *bxd*PRE silencing at the four other attP sites which do not have peaks for Su(Hw) nearby (Additional file 3). To control for the effects of the Su(Hw) binding sites on transcriptional activity, the Su transgene was inserted at these other attP sites as well. Shown in Fig. 3 is a comparison of the silencing activity of the *bxd*PRE with and without the 4xSu(Hw) multimer (Fig. 3a,b) and 4xSu(Hw) multimer alone (Fig. 3c). For inserts at 22A, 51C, and 58A, silencing of *white* in hemizygotes is enhanced when 4xSu(Hw) is included next to *bxd*PRE in the transgene (Fig. 3b). While there is no obvious effect on the silencing of inserts at 68E (Fig. 3b), the *bxd*PRE already strongly silences on its own as either a hemizygote or homozygote (Fig. 3a). In homozygotes, the 22A insertion containing 4xSu(Hw) and *bxd*PRE is lethal, while the PSS observed for the 58A insert is clearly stronger when the 4xSu(Hw) multimer is present (Fig. 3b). Although there is no obvious difference between hemizygotes and homozygotes for the Su-bxd transgene inserted at 51C, silencing is substantially greater when the 4xSu(Hw) multimer is present (Fig. 3b). For the control construct, the Su(Hw) multimer alone, there is no evidence for silencing in any of these lines either in hemi- or homozygotes (Fig. 3c). Taken together, these findings indicate that the 4xSu(Hw) multimer can augment the silencing activity of the *bxd*PRE in different chromosomal environments.

### Binding sites for architectural proteins CTCF and Pita can induce *bxd*PRE silencing

Next, we wondered whether induction of PRE repressing activity is unique to Su(Hw) or whether other polydactyl zinc finger proteins that have chromosome architectural functions are able to induce PRE silencing. To test this, we linked the *bxd*PRE to either a *Drosophila* 4xCTCF multimer or a 5xPita multimer. Like 4xSu(Hw), both of these multimers have insulating activity in BX-C boundary replacement experiments [71, 75–77]. Figure 4 shows that combining either 4xCTCF or 5xPita with the *bxd*PRE induces silencing activity in hemizygotes and PSS in homozygotes. Control experiments show that *white* expression is not affected when the multimers are included in the transgene alone. Thus, multimerized sites for three different chromosomal architectural proteins can induce the silencing activity of the *bxd*PRE.

### Su(Hw) architectural protein induces *en*PRE silencing activity

**Fig. 5:**
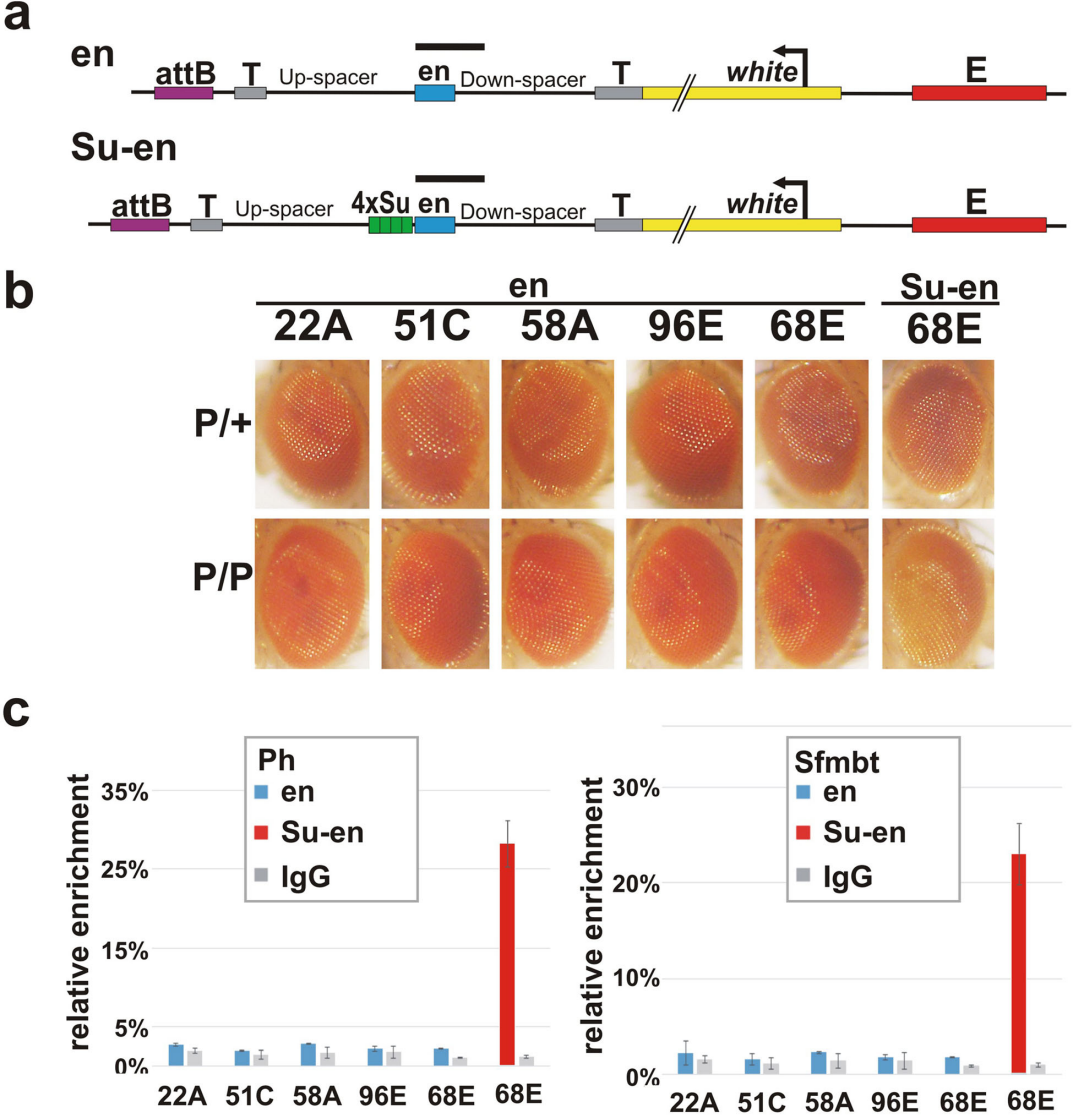
Su(Hw) multimer can induce *en*PRE silencing. **a** Map of “en” and “Su-en” transgenes. Designations: “*en*”—*en*PRE element. Other labels are as in Fig. 1. **b** The eye phenotypes of flies with “en” and “Su-en” transgenes integrated into different attP insertion sites. **c** The results of X-ChIP with chromatin isolated from adult heads of homozygous lines with “en” or “Su-en” transgenes integrated into different attP insertion sites. X-ChIP was performed with Ph and Sfmbt antibodies or with an IgG control followed by real-time PCR with primers specific to transgenic *en*PRE. The ordinate shows the percentage of target sequences in the immunoprecipitated material relative to the input DNA and normalized relative to *bxd*PRE-Genome. The lines with *en*PRE transgene are designated by the attP insertion site as indicated on the abscissa. Other designations are as in Fig. 2

We also used the same strategy to test the silencing activity of another well-defined PRE, the 181 bp *en*PRE (PSE2) from the *engrailed* locus, in different chromosomal environments. In previous P-element transgene experiments, this element was shown to repress *white* expression as a hemizygote and/or homozygote and maintain the parasegmental expression pattern of a *Ubx* reporter [33, 38, 46, 59, 78–82]. Surprisingly, however, repression of *white* by the *en*PRE in hemizygote flies is weak or nonexistent at all five attP insertion sites and there is no evidence of PSS when the inserts are homozygous (en construct, Fig. 5a, b) Moreover, Ph binding is not detected at the *en*PRE in homozygous insertions of this construct (Fig. 5c).

Since the lack of silencing was unexpected, we tested whether silencing activity could be induced by linking the 4xSu(Hw) multimer to the *en*PRE (Su-en construct, Fig. 5a). For this purpose, we chose the 68E attP integration site as it was most permissive for *bxd*PRE silencing. Figure 5b shows that the eye pigmentation of hemizygous Su-en flies is similar to that observed for en flies; however, silencing is observed in flies homozygous for the 68E insert. This is opposite of that observed for the en transgene at 68E where the eye color in homozygotes becomes darker not lighter. ChIP experiments provide further evidence that 4xSu(Hw) is able to activate PcG-dependent silencing. While Ph and Sfmbt are not associated with the *en*PRE in the en transgene, both are recruited to the *en*PRE when it is linked to 4xSu(Hw) (Fig. 5c).

### Increasing the distance between the *bxd*PRE and the boundary element disrupts silencing in hemizygotes

How do the architectural proteins help establish PRE activity? Two different mechanisms could be in play. The architectural proteins could facilitate the establishment of PcG silencing by locally displacing nucleosomes and/or by recruiting chromatin remodeling complexes so that key DNA-binding factors and PcG complexes are able to assemble on the PRE. Alternatively, the architectural proteins could enhance PRE activity by helping target the transgene from an active chromosomal compartment to a PcG-silenced chromosomal compartment [83]. Since the effects of nucleosome displacement and chromatin remodeling are expected to be limited to closely linked sequences, the former model predicts that the impact of architectural proteins on the establishment of PcG silencing will decrease as the distance between the multimers and the *bxd*PRE is increased. In the latter model, relatively small changes in distance (<10 kb) should have little or no effect on the ability of the boundary to target the transgene to a PcG-silenced compartment.

**Fig. 6:**
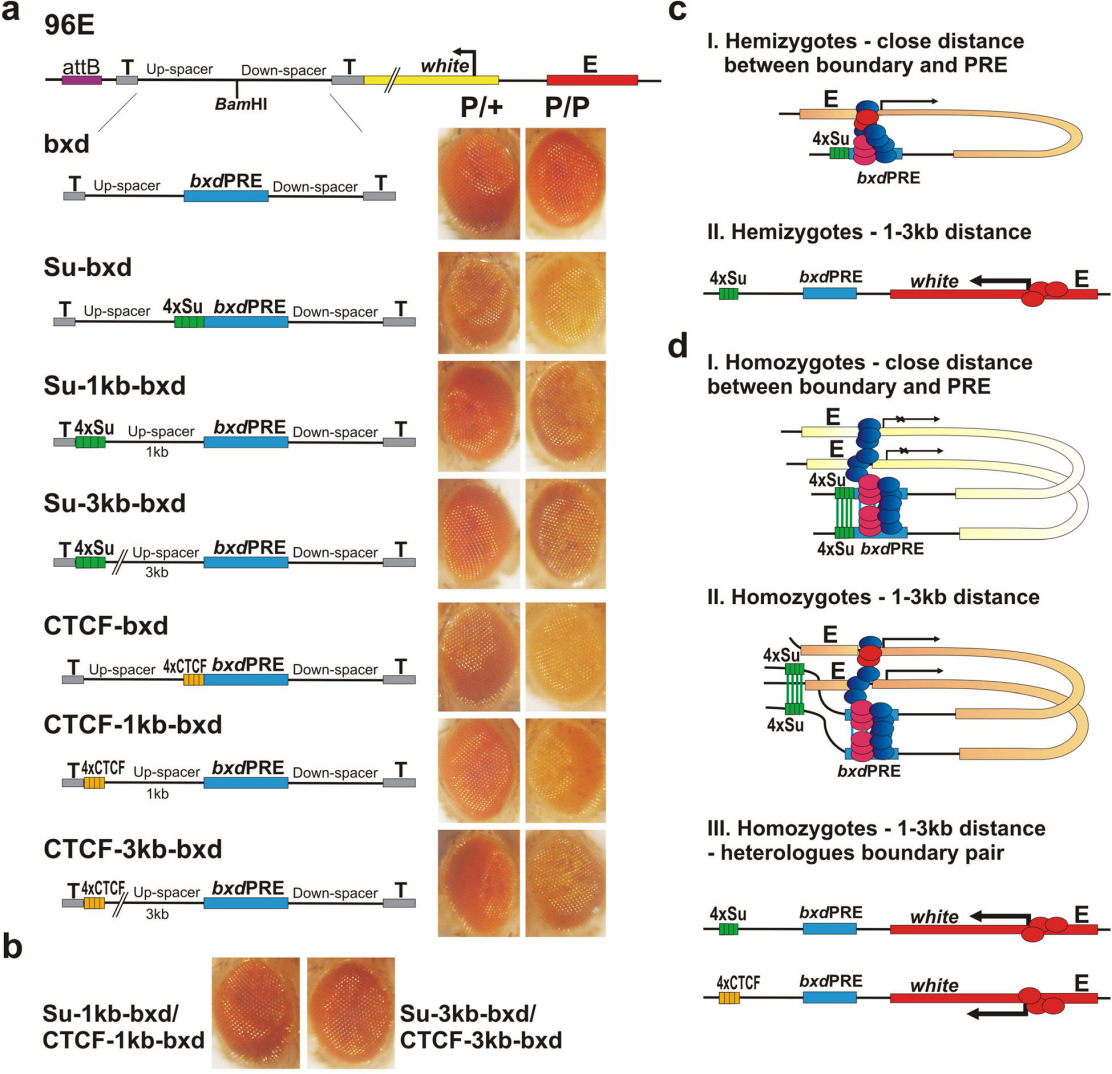
Impact of distance between the boundary and the *bxd*PRE on PcG-dependent silencing. All transgenes were integrated in 96E insertion site. **a** Su-1kb-bxd, Su-3kb-bxd—the 4xSu sites are placed at the distance of 1 or 3 kb from the *bxd*PRE. CTCF-1kb-bxd, CTCF-3kb-bxd—the 4xCTCF sites are placed at the distance of 1 or 3 kb from the *bxd*PRE. Eye phenotypes in hemizygous (P/+) and homozygous (P/P) flies are shown. **b** The phenotypes of *trans*-heterozygotes. To generate the Su-1kb-bxd/CTCF-1kb-bxd or Su-3kb-bxd/CTCF-3kb-bxd *trans*-heterozygotes, Su-1kb-bxd and CTCF-1kb-bxd or Su-3kb-bxd and CTCF-3kb-bxd homozygous flies were crossed with each other. Hypothetical models of interactions in hemizygotes (**c**), homozygotes, and *trans*-heterozygotes (**d**) are shown. Red ovals—enhancer-associated activator proteins; blue and pink ovals—Polycomb and Trithorax group proteins, respectively

To test these two models, we increased the distance between the 4xSu(Hw) or 4xCTCF multimers and the *bxd*PRE by 1 kb and 3 kb (Fig. 6a). For the distance of 1 kb, we inserted each multimer upstream of the left eGFP coding sequence spacer (the Su-1kb-bxd and CTCF-1kb-bxd constructs). To adjust the distance between the multimers and the PRE to 3 kb, we introduced an additional 2 kb spacer derived from the *Escherichia coli LacZ* coding sequence (the Su-3kb-bxd and CTCF-3kb-bxd constructs). A 1-kb distance would be sufficient for approximately five nucleosomes, while 3 kb would correspond to about fifteen nucleosomes. All of the constructs were then introduced into the 96E attP site. The results of this analysis are shown in Fig. 6. In hemizygotes (P/+) increasing the distance between the multimer and the *bxd*PRE adversely impacts silencing activity. For the 4xSu(Hw) multimer, a distance of 1 kb is sufficient to substantially reduce silencing (compare with *bxd*PRE alone). In the case of the 4xCTCF multimer, the disruption of silencing is greater when it is located 3 kb away from the *bxd*PRE than it is at a 1-kb distance; however, even at 1 kb, silencing is reduced compared to the control CTCF-bxd construct. These findings argue in favor of the first model, namely that the 4xSu(Hw) and 4xCTCF multimers act in *cis* to facilitate the assembly of silencing complexes on the PRE (Fig. 6c).

### Boundaries located at a distance from the bxdPRE can induce PSS

While silencing activity in hemizygotes is significantly compromised when the multimers are moved away from the *bxd*PRE, this is not true for PSS. As shown in Fig. 6 (P/P), the *bxd*PRE represses *white* expression even when the boundary multimers are located 3 kb away. A plausible explanation for this result is that pairing of the 4xSu(Hw) or 4xCTCF multimers in *trans* would tend to stabilize pairing interactions between the PREs on each homolog, and this interaction facilitates the PSS-dependent assembly of functional silencing complexes.

Boundary pairing in *Drosophila* depends upon specific interactions between proteins associated with each element [56, 84–86]. A classic example of specificity comes from the boundary bypass assay. In this assay, an upstream regulatory element (enhancer or silencer) is separated from a reporter gene by a spacer DNA that is flanked by two boundary elements (endogenous or artificial). If the boundaries flanking the spacer DNA can pair with other, the upstream regulatory element is brought into close proximity with the reporter and can either activate (enhancer) or repress (silencer) its expression [48, 55, 57, 87, 88]. This is what is found when the spacer DNA is flanked by either Su(Hw) or CTCF multimers [81]. On other hand, bypass is not observed when the spacer DNA is flanked by a heterologous combination of Su(Hw) multimers and CTCF multimers [89]. Thus, if boundary-boundary interactions between homologs are required to induce PRE repression, then PSS should not be observed in the two sets of mixed pairs: Su-1kb-bxd *trans* to CTCF-1kb-bxd or Su-3kb-bxd *trans* to CTCF-3kb-bxd. Figure 6b shows that this prediction is correct: silencing depends on boundary pairing in *trans* and is not observed in heterologous combinations. A possible model is shown in Fig. 6d.

### Binding sites of architectural proteins leads to local decrease of histone H3 enrichment

**Fig. 7:**
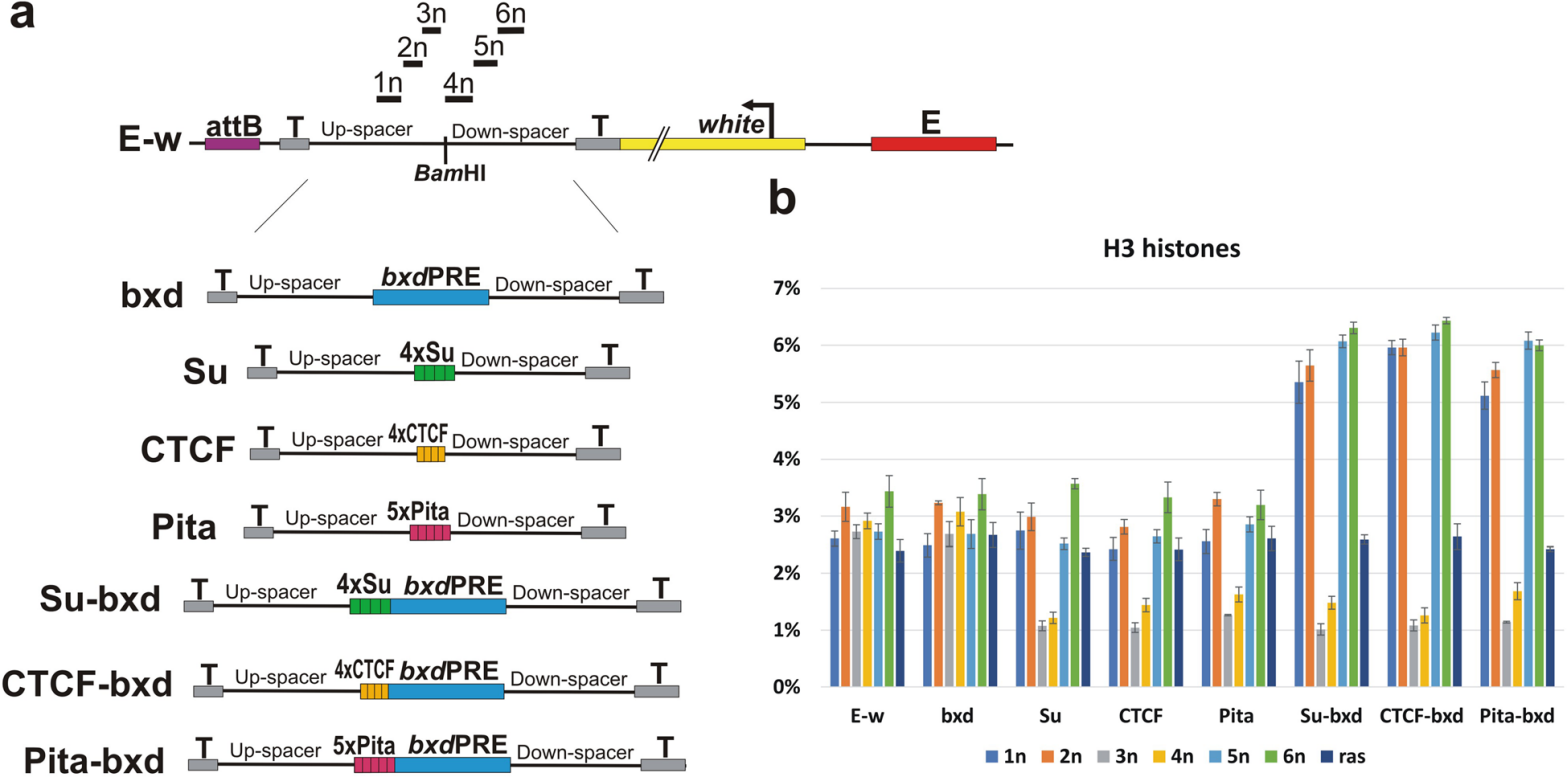
Multimerized binding sites for the three polydactyl zinc finger architectural proteins induces a local reduction in histone H3 occupancy. **a** Maps of transgene constructs used for the histone H3 ChIPs: E-w, bxd, Su, CTCF, Pita, Su-bxd, CTCF-bxd, Pita-bxd. The location of the sequences amplified by qPCR in X-ChIP experiments are indicated above the E-w construct map (1n, 2n, 3n, 4n, 5n, 6n). They are the same for all of the constructs tested. **b** X-ChIPs were performed with chromatin isolated from heads of adult flies homozygous for transgenes inserted at 96E. X-ChIPs were performed with histone H3-specific antibodies or with the IgG control. The ordinate shows the percentage of target sequences in the immunoprecipitated material relative to the input DNA. The abscissa indicates the transgene construct and amplified primer pair. The ras (coding part of Ras64B gene) was used as an internal control

The finding that the boundary element must be closely linked to the *bxd*PRE for silencing in hemizygotes and enhanced PSS in homozygotes suggests that the boundary has a local effect on chromatin structure that enables PcG factors to gain access to the PRE and assemble functional silencing complexes. If this suggestion is correct, then the chromatin organization of the *bxd*PRE should be altered when it is closely linked to a boundary element. To test this prediction, we analyzed histone H3 association with six unique sequences (1n-6n) located at different distances upstream and downstream of the BamH1 site used to insert test DNAs (Fig. 7a). The distance between the midpoints of the 3n and 4n sequences and the BamH1 site are 75 bp and 83 bp, respectively. The midpoints of 2n and 5n are 187 bp and 233 bp from the BamH1 site, respectively, while those for 1n and 3n are 301 and 365 bp.

In the control E-w transgene, histone H3 association as measure by ChIP is nearly the same in all regions tested (1n-6n) and is equivalent to that observed for the control genomic sequence in the coding region of the Ras64B gene (ras) (Fig. 7b). The inclusion of the *bxd*PRE in the transgene (bxd) has no apparent effect on histone H3 occupancy and the profile across the six sequences is similar to that of the E-w control. A different result is obtained for Su, CTCF, or Pita transgenes containing the multimerized binding sites for the boundary proteins. In all three cases, there is a reduction in histone H3 occupancy in the sequences located immediately next to the multimers (Fig. 7b). This finding indicates that the multimerized sites for these three chromosomal architectural proteins generate a region that is depleted in nucleosomes. This effect is local and does not extend to sequences located more distant (1n, 2n, 5n, and 6n) from the multimerized sites.

We next examined the Su-bxd, CTCF-bxd, and Pita-bxd transgenes (Fig. 7b). As was observed for the transgenes containing only the multimerized binding sites, histone association with the two sequences, 3n and 4n, that immediately flank the multimer-*bxd*PRE combination is reduced. In each case, the reduction is approximately the same as that observed for the corresponding multimer alone. Importantly, this is true for 4n, which is separated from the multimers by the 650 bp *bxd*PRE. These results provide strong support for the idea that closely linked boundary elements can induce alterations in the association of nucleosomes with the PRE. Interestingly, this is not the only alteration in nucleosome association evident in these transgenes. In all three of the multimer-*bxd*PRE combinations, we found that histone H3 association with the more distant sequences (1n, 2n, 5n, and 6n) is enhanced relative to the various control transgenes (E-w, bxd and Su, CTCF, and Pita). Since enhanced association is not observed with the inactive *bxd*PRE or with any of the multimers alone, it seems likely that this effect is due to the recruitment of functional PcG complexes to the *bxd*PRE. It remains to be determined whether this alteration in histone H3 association reflects an increase in nucleosome density in the flanking DNA regions or an increase in the extent of compaction.

### Interplay between PREs and boundary contributes to silencing

The studies in the previous sections indicate that boundaries can enhance PRE silencing by two different mechanisms. One takes place in *cis* and requires a close linkage of the boundary and PRE. This mechanism locally alters the pattern of histone association and facilitates the recruitment of factors critical for PcG repression. The other takes place in *trans* and is mediated by boundary:boundary pairing interactions. In this second mechanism, boundaries appear to provide a “spot weld” that holds the homologs in close proximity. This facilitates PRE:PRE interactions and results in PSS even when the boundary multimers are separated from the PREs. We undertook several additional experiments to further explore this “pairing” mechanism.

**Fig. 8:**
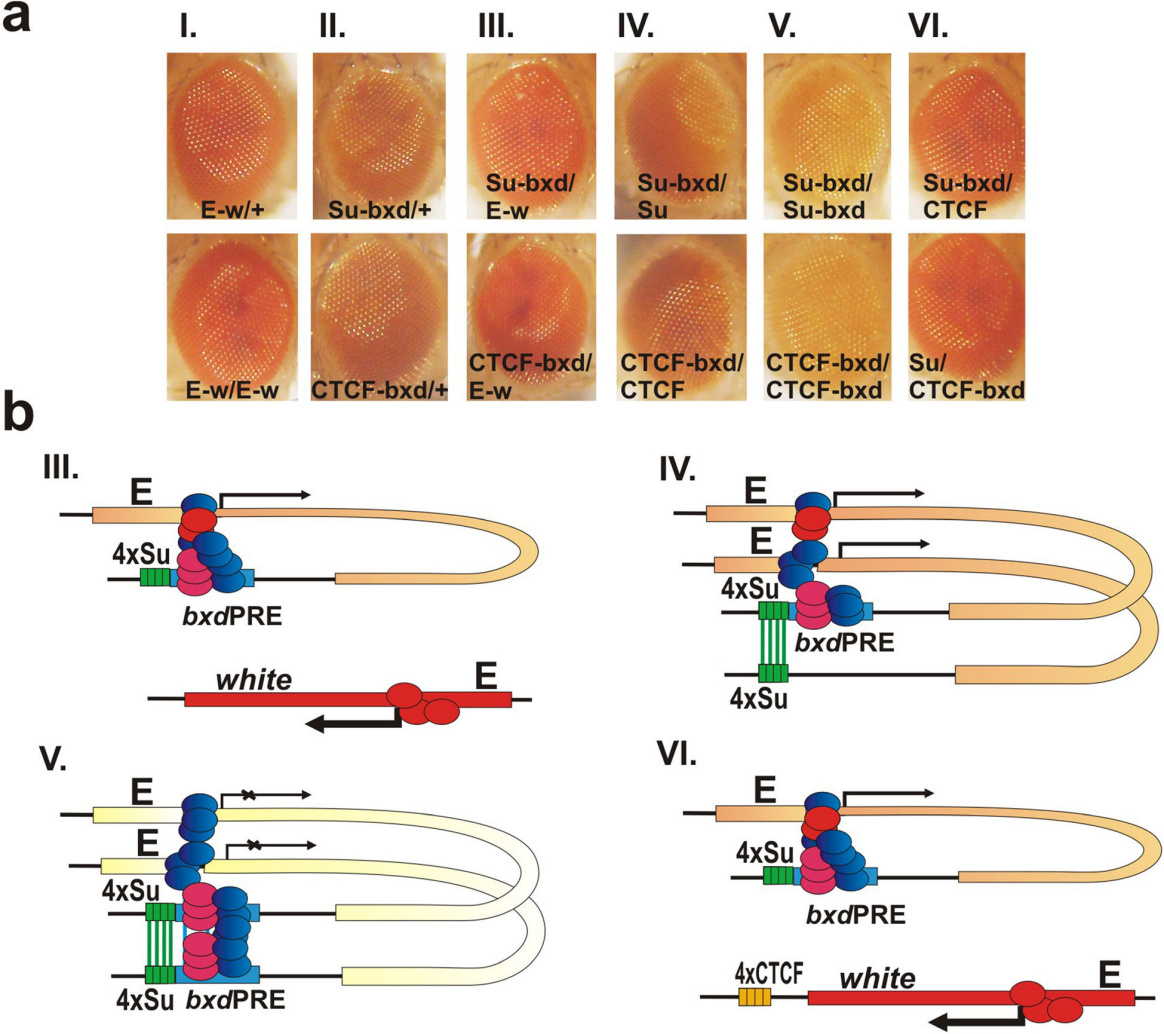
Induction of PRE silencing in *trans* by boundary:boundary pairing. **a** Phenotypes of eyes of transgenic flies. *Trans*-heterozygotes were obtained by crossing the corresponding homozygote flies. All transgenes used for crosses were integrated at 96E. **b** III, IV, V, VI—hypothetical chromatin interactions formed in a-III, a-IV, a-V, a-VI, respectively

*Boundary pairing can induce silencing in trans*: When 4xSu(Hw) or 4xCTCF is placed next to the *bxd*PRE, the PRE can assemble a functional silencing complex and repress *white* in hemizygotes. However, the *bxd*PRE would not be expected to efficiently silence *white* in *trans* unless the homologs are tightly paired in the immediate neighborhood. To test this expectation, we generated *trans*-heterozygotes between the starting transgene E-w (the eye enhancer—*white* gene control construct which has all of the elements in the bxd construct except bxdPRE) and either Su-bxd or CTCF-bxd (Fig. 8a). While the *bxd*PRE silences the *white* gene in *cis* (Fig. 8a-II, Su-bxd/+ and CTCF-bxd/+ - compare with E-w/+ in Fig. 8a-I), it does not efficiently silence the *white* gene in *trans* (Fig.8a-III, Su-bxd/E-w, and CTCF-bxd/E-w: compare with E-w/+ and E-w/E-w in Fig. 8a-I). In these two combinations, the eye color phenotype is indistinguishable from that observed in E-w /+ or E-w/E-w. A different result is obtained when both transgenes have a copy of the same multimer (Su-bxd/Su or CTCF-bxd/CTCF, Fig. 8a-IV—compare with Su-bxd/E-w and CTCF-bxd/E-w, Fig.8a-III). While silencing is not as effective as when the two transgenes not only have identical multimers but also a copy of the *bxd*PRE (see Fig. 8a-V, Su-bxd/Su-bxd, CTCF-bxd/CTCF-bxd), the level of *white* expression is clearly reduced compared to that observed when the multimer is not present in the E-w transgene. This finding indicates that boundary:boundary pairing interactions can promote *trans* silencing by a PRE. To confirm that pairing interactions between the boundaries in the two transgenes provide a “spot weld” that facilitates *trans* silencing activity, we tested heterologous multimer combinations that do not pair with each other. As shown in Fig. 8a-VI, silencing is not observed with the Su-bxd/CTCF combination, nor is it observed with the converse CTCF-bxd/Su combination.

**Fig. 9:**
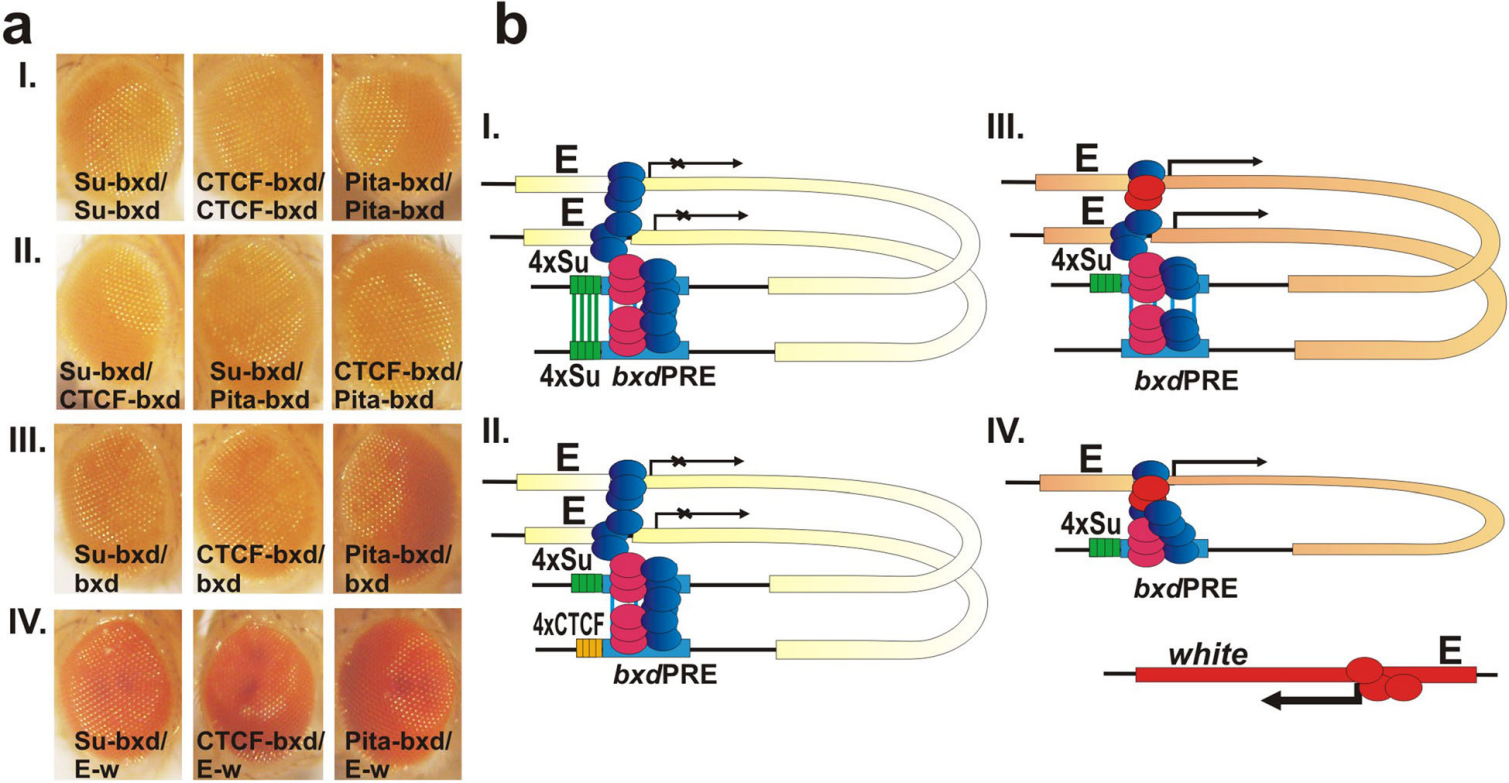
PRE dependent *trans*-activation of silencing. All transgenes used for crosses were integrated in 96E insertion site. **a** Eye phenotypes of transgenic flies. **b** I, II, III, IV—the hypothetical chromatin interactions formed in a-I, a-II, a-III, a-IV, respectively

*Boundary pairing is not necessary when both PREs are active:* Placing multimerized binding sites for zinc finger architectural proteins next to the *bxd*PRE induces the assembly of functional silencing complexes in hemizygous flies and enhances PSS in homozygous flies. If the PREs on both homologs are activated by multimerized binding sites, then boundary:boundary pairing interactions would not be expected to be required for generating the synergistic *trans* interactions between PREs on each homolog that are responsible for PSS. To test this prediction, we generated *trans* combinations of *bxd*PRE transgenes that have closely linked multimerized Su(Hw), CTCF, or Pita-binding sites. Figure 9a-II shows that the silencing of *white* in the three *trans*-heterologous combinations of multimerized binding sites is close to that observed when both transgenes have multimerized binding sites for the same protein.

*An inactive PRE can be transactivated by an active PRE*: The *bxd*PRE insertion in the 22A attP site is a classic example of PSS. Little or no silencing is observed in hemizygotes, while silencing is quite efficient in homozygotes. The PSS phenomenon suggests that PRE:PRE interactions in *trans* can synergizes, promoting the assembly of functional PcG silencing complexes on both PREs. To test this idea for 96E, we generated *trans* combinations between the inactive PRE in the bxd transgene and transgenes in which *bxd*PRE is activated by closely linked 4xSu(Hw), 4xCTCF, or 5xPita sites. Consistent with prediction, the *white* reporter in the homolog carrying the *bxd*PRE (only) transgene is repressed when the other homolog has the boundary-*bxd*PRE (compare eyes in Fig. 9a-III with those in Fig. 9a-IV). However, silencing is not equivalent to that observed when the *bxd*PREs on both homologs are activated by multimerized binding sites (Fig. 9a-I).

## Discussion

PcG-dependent silencing is widely used during the development of multicellular organisms as a mechanism for maintaining the determined state [4–10]. In flies, PREs are responsible for recruiting the PcG complexes, PRC1, PRC2, and PhoRC, to repress transcription of their target genes [26–29]. Since PREs are able to establish and maintain silencing at ectopic locations in transgene assays, it is possible to study some of the parameters that govern their activities. In order to better control the chromosomal context, we selected five attP sites (22A, 51C, 58A, 68E, and 96E) that appear to be “neutral” with respect to their effects on *white* expression. Unexpectedly, in spite of this apparent homogeneity, the silencing activity of the *bxd*PRE differs substantially from one site to the next. At one extreme (68E), strong silencing of *white* is observed in hemizygotes, while at the other extreme (96E) there is no evidence of silencing even in homozygotes. For the three remaining attP sites, there is a range of silencing in hemizygous and homozygotes. For 22A and 58A, there is little evidence of silencing in hemizygous flies, while PSS is observed in homozygotes. In the case of 51C, *white* expression is clearly repressed in hemizygotes, and repression increases in homozygotes. The strength of silencing at the five attP sites correlates well with the recruitment of the PcG protein Ph. High levels are found associated with *bxd*PRE when it is inserted at 68E, while only background levels are evident at 96E.

These findings are consistent with previous P-element transgene-based experiments, which showed that the silencing activity of PREs is non-autonomous and depends upon the local chromosomal context [42, 44–46]. The fact that Ph is not detected at 96E suggests that the establishment of silencing depends upon whether DNA binding proteins and PcG complexes can gain access to the PRE sequences at the site of the transgene insertion. Consistent with this idea, we found that like Ph several key PcG proteins are not associated with the *bxd*PRE insert at 96E.

These findings led us to wonder what features of the chromatin landscape in the neighborhood of the PRE might facilitate the recruitment of the required silencing factors. In this respect, it was of interest that PREs in BX-C are often closely linked to chromatin boundary elements [1]. This correlation suggested that boundary elements might be able to mediate the reorganization of the chromatin so that key silencing factors can gain access to the PRE. We tested this possibility by placing multimerized binding sites for three different chromosomal architectural proteins, Su(Hw), CTCF, and Pita, adjacent to the *bxd*PRE. We found that all three of these artificial boundaries were able to induce silencing in hemizygotes and PSS in homozygotes. For the 4xSu(Hw)-*bxd*PRE combination, we found that the acquisition of silencing activity is accompanied by the recruitment of both components of the PhoRC DNA binding complex, Pho and Sfmbt, and other PcG silencing factors to the *bxd*PRE. Surprisingly, we found that two TrxG proteins, Trx and CBP, are also associated with the *bxd*PRE, and like the PcG factors, this association is only observed when the *bxd*PRE is closely linked to the 4xSu(Hw) multimer.

In hemizygotes, silencing activity is induced when the boundary multimers are placed next to the *bxd*PRE. Induction of silencing is distance dependent, and for the 4xSu(Hw) multimer, a distance of 1 kb is sufficient to largely eliminate silencing in hemizygotes, while it is reduced but not entirely eliminated for the 4xCTCF multimer. The importance of proximity taken together with the de novo recruitment of DNA binding and PcG proteins suggests that the boundary might alter the local chromatin structure so that factors important for PcG-dependent silencing can gain access to the PRE. Consistent with this hypothesis, we find that multimerized binding sites for the boundary factors Su(Hw), CTCF, and Pita can, on their own, generate a local reduction in histone H3 occupancy, indicating that the multimers likely form a nucleosome-free region of chromatin like that observed for many endogenous boundaries [90–92]. When the *bxd*PRE is placed next to these multimers, the nucleosome free region extends across the 650-bp *bxd*PRE fragment. While we did not investigate the mechanisms of nucleosome displacement, Su(Hw) and CTCF have been shown to interact with chromatin remodeling complexes. Su(Hw) was found to recruit the Brahma chromatin remodeling protein [92], while both Su(Hw) and CTCF co-immunoprecipitate subunits of the NURF remodeling complex [93, 94]. Moreover, when CTCF is targeted to polytene chromosomes via linkage to the GAL4 DNA-binding domain, local chromatin decondensation is observed [95]. It would be reasonable to think that recruitment of these remodeling factors to the boundary multimers would in turn facilitate the displacement of nucleosomes from the PRE, enabling PcG (and TrxG) factors to gain access. Interestingly, activation of PcG silencing results in an increase in nucleosome density in the sequences flanking the PRE and the multimers. Since this phenomenon is not observed with the multimers alone, it seems possible that this change in nucleosome organization may be linked to PcG-dependent silencing.

Consistent with idea that the boundary multimers facilitate the displacement of nucleosomes from the PRE, we find that silencing activity is compromised when the multimers are placed at a distance of 1 to 3 kb from the PRE. This distance effect would seem to argue against a mechanism in which the multimers target the PRE to a PcG compartment. On the other hand, while the boundary multimers are unable to fully augment silencing activity in hemizygotes unless they are next to the *bxd*PRE, the presence of multimers in the transgene even at a distance of 3 kb is sufficient to promote PSS when the transgene is homozygous. Critically, the induction of PSS in this configuration is dependent on the ability of the boundaries on each homolog to pair with each other as it is not evident when the 4xSu(Hw) multimer is *trans* to the 4xCTCF multimer. A plausible interpretation of this finding is that when inactive PREs on each homolog are brought into close proximity by the paired boundaries, there is a synergistic interaction, which is able to promote the acquisition of PcG-dependent silencing activity. This model predicts that an active PRE should be able to *trans*-activate an inactive PRE. Consistent with this prediction, we found that an active *bxd*PRE (linked to a multimer) on one homolog can partially *trans-*activate an inactive *bxd*PRE (unlinked to a multimer) on the other homolog.

## Conclusions

Here, we show that boundary elements can help induce the silencing activity of PREs by facilitating the recruitment of DNA-binding and PcG/TrxG proteins. Unlike the *bxd*PRE, the artificial boundaries we tested are able to autonomously establish regions of chromatin that have reduced nucleosome occupancy. When the *bxd*PRE is placed next to one of these multimers, the region of reduced nucleosome occupancy extends over the PRE. Accompany this reduction in occupancy, PcG and TrxG factors are found associated with the *bxd*PRE and silencing activity is observed. In addition to this local effect on PcG silencing activity, we found that boundaries can induce PSS when placed at a distance from the PRE. In this case, our genetic experiments indicate that boundary:boundary pairing forms a “spot weld” which enables the PREs on each homolog to interact with each other and induce PcG silencing.

## Methods

### *Drosophila* strains, germline transformation, and genetic crosses

All flies were maintained at 25°C on the standard yeast medium. The constructs were injected into embryos of lines ZH-attP-22A (BDSC #24481), ZH-attP-51C (BDSC #24482), ZH-attP-58A (BDSC #24484), ZH-attP-68E (BDSC #24485), and ZH-attP-96E (BDSC #24487) [60]. The resulting flies were crossed with *yacw*^*1118*^ flies, and the transgenic progeny was identified by their eye pigmentation. Details of crosses used for genetic analysis are given in Additional file 4.

For phenotype analysis of *white* expression, we visually determined the degree of pigmentation in the eyes (*white*) of 3- to 5-day-old males developing at 25°C, with reference to standard color scales. Pigmentation of all flies was analyzed in hemi- (P/+) or homozygotes (P/P).

### Plasmid construction

The *Bam*HI-*Eco*RI fragment containing the *white* gene (*mini-white* version) without Wari insulator was clones from pCaSpeR∆700 vector [96] into the pBluescript SK+ vector to obtain white-rev-pSK plasmid. The attB site was PCR-amplified with 5′-gtcgacgatgtaggtcacgg-3′ and 5′-gtcgacatgcccgccgtgac-3′ primers and cloned downstream of the *white* gene (attBdir-*white*-rev-pSK). The sequence corresponding to the eye enhancer (E) of the *white* gene (regulatory sequences from position –1180 to –1849 bp relative to the transcription start site) was cut out of the Ee− pBluescript SK+ plasmid [63] without flanking sequences and cloned in direct orientation upstream of the *white* gene (attBdir-*white*-rev-E-rev-pSK).

The coding regions of eGFP (PCR-amplified with primers 5′-atggtgagcaagggcgaggagct-3′ and 5′-cttgtacagctcgtccatgccga-3′) and Cherry (PCR-amplified with primers 5′-atggtgagcaagggcgaggag-3 and 5′-ttacttgtacagctcgtccat-3′) were cloned into pBluescript SK+ vector in a “head-to-head” orientation (eGFPrev-Cherry-pSK). The SV40 and *yellow* gene terminators of transcription were cloned downstream eGFP and Cherry respectively in orientation to stop the transcription from flanking regions (SV40dir-eGFPrev-Cherrydir-Ytermrev).

To obtain E-w construct, the fragment corresponding to SV40dir-eGFPrev-Cherrydir-Ytermrev was cloned into attBdir-*white*-rev-E-rev-pSK plasmid between attB and the *white* gene. The resulting E-w construct has Bam*HI* cloning site between the eGFP and RFP coding regions that was used to create the following constructs. The 656bp *bxd*PRE (corresponding to the genome sequences between *Pst*I-*Nde*I) was PCR amplified from the frt(PRE) plasmid [97] with direct primer (5′-aaaagatctctcgagaaactagtgaggcagcgactgcgc-3′ having *Bgl*II, Xho*I*, and Spe*I* restriction sites) and reverse primer (5′-tttggatccgatagcttgatgatccaac-′ having additional *Bam*HI site). The *bxd*PRE PCR fragment was then cleaved by *Bam*HI-*Bgl*II and cloned into E-w cleaved by *Bam*HI (bxd construct). The 4xSu(Hw) sites (4xSu) were cut from a plasmid used previously [98] and cloned into the bxd construct cleaved by Xho*I* and Spe*I* (Su-bxd construct) or into the E-w construct cleaved by *Bam*HI (Su construct). CTCF-bxd, CTCF, Pita-bxd, Pita, en, and Su-en constructs were generated by the same scheme using the following plasmids and fragments. The plasmids with 4xCTCF [75] and 5xPita [76] sites were kindly provided by Olga Kyrchanova. A 188-bp fragment containing the enPRE was PCR-amplified using 5′-gagatggcatgtggctctc-3′ and 5′-attatgcgcatgctggagctgtc-3′ primers. To create Su-1kb-bxd, CTCF-1kb-bxd constructs, the Sph*I* restriction site was inserted upstream of eGFP (SphI-bxd). The 4xSu fragment or 4xCTCF was inserted into SphI-bxd cleaved by Sph*I*. To create Su-3kb-bxd, CTCF-3kb-bxd constructs, the 2087-bp fragment containing the LacZ coding region was inserted into Su-1kb-bxd, CTCF-1kb-bxd constructs between 4xSu and eGFP. The sequences corresponding to construct fragments (attB, SV40 terminator, eGFP, 4xSu, 4xCTCF, 5xPita, *bxd*PRE, *en*PRE, Cherry, Yterm, *white*, and E) are given in Additional file 4.

### Chromatin immunoprecipitation (X-ChIP)

For each experiment, 150–200 mg of heads from 2- to 5-day-old adults homozygous for the different constructs were collected. Experimental procedures for chromatin immunoprecipitations were performed as described previously [62, 63]. Briefly, the material was homogenized in 5 ml of buffer A1 (15 mM HEPES, pH 7.6; 60 mM KCl, 15 mM NaCl, 4 mM MgCl2, 0.5% Triton X-100, 0.5 mM DTT) supplemented with the EDTA-free protease inhibitor cocktail (Roche, Switzerland) and formaldehyde as a crosslinking agent (final concentration 1.8%). The reaction was stopped by adding glycine (final concentration 225 mM). The homogenate was cleared by passing through 100-μm nylon cell strainer (BD Falcon) and pelleted by centrifugation at 4000 g, 4°C for 5 min. After washing in three 3-ml portions of buffer A1 at 4°C (5 min each) and 3 ml of lysis buffer without SDS, the pellet was treated with 0.5 ml of complete lysis buffer (15 mM HEPES, pH 7.6; 140 mM NaCl, 1mM EDTA, 0.5 mM EGTA, 1%Triton X-100, 0.5 mM DTT, 0.1% sodium deoxycholate, 0.1% SDS, 0.5% *N*-lauroylsarcosine, EDTA-free protease inhibitor cocktail) and sonicated to break chromatin into fragments with an average length of 700 bp. The material was pelleted by centrifugation at 18 000 g for 5 min, and the supernatant fluid was transferred to a new tube. The pellet was treated with the second 0.5-ml portion of lysis buffer, and the preparation was centrifuged at 18 000*g* for 5 min. The two portions of the supernatant fluid were pooled, cleared by centrifuging twice at 18 000*g* for 10 min, and the resultant chromatin extract (1 ml) was used for ChIP experiments after preincubation with A-Sepharose or G-Sepharose. One aliquot (1/10 volume) of chromatin extract after preincubation with Sepharose was kept as a control sample (Input).

ChIP experiments involved incubation with specific rabbit/rat antibodies or with nonimmune IgG that was used as nonspecific antibody control. All ChIP experiments were made at least in triplicate. The enrichment of specific DNA fragments was analyzed by real-time qPCR, using a C1000™ Thermal Cycler with CFX96 real-time PCR detection module (Bio-Rad) or a StepOne Plus Thermal Cycler (Applied Biosystems, USA). Primers used in ChIP/real-time PCR analyses are listed in Additional file 1: Table S2 and Table S3.

### Antibodies

Antibodies against Ph (86-520 aa, ph-p, isoform PA), Sfmbt (1-348 aa of isoform PB) [62, 63], and Trx-N (8-351 aa of isoform PA) [62] were raised in rabbits and described previously. Antibodies against Combgap (31-269aa of isoform PF) and CBP (1-290 aa of isoform PB) were raised in rabbits. Pho (full length of isoform PA) was raised in rats. Antigens for antibody production were expressed as a 6×His-tagged fusion proteins in *Escherichia coli*, affinity purified on Ni Sepharose 6 Fast Flow (GE Healthcare) according to the manufacturer’s protocol and injected into rabbits following the standard immunization procedure. Antibodies were affinity-purified from serum on the same antigen as was used for immunization. The specificity of Combgap, CBP, and Pho antibodies was verified by RNAi/Western blotting (Additional file 1: Figure S2) according to protocol described previously [99]. Anti-lamin ADL67.10 antibodies were provided by the Developmental Studies Hybridoma Bank (deposited to the DSHB by Fisher, P. A., DSHB Cat# adl67.10, RRID:AB_528336). For nucleosome, ChIP commercial antibodies (Abcam Cat# ab1791, RRID:AB_302613) were used.

## Supplementary Information

**Additional file 1: Table S1.** Eye phenotypes of hemizygote transgene flies with insertion of *white* marker gene without enhancer at selected attP lines. **Figure S1.** X-ChIP for the “Su” transgene with Ph antibody and X-ChIP for the “Su-bxd” and “Su” transgenes with Su(Hw) antibody. **Table S2.** Primers used for X-ChIP-qPCR analysis to test PcG/TrxG, DNA-binding proteins recruitment to transgenes. **Table S3.** Primers used for X-ChIP-qPCR analysis to test histone H3 binding to transgenes. **Figure S2.** Antibody specificity test.

**Additional file 2.** This file contains the analysis of homologue chromosomes contact frequencies at the 22A, 51C, 58A, 68E and 96E attP insertion sites.

**Additional file 3.** This file contains the ChIP-seq data for PcG and boundary proteins binding to 22A, 51C, 58A, 68E and 96E genome regions.

**Additional file 4 **This file contains: **(1)** the DNA sequences of the construct elements. **(2)** the details of *Drosophila* genetic crosses.

## Data Availability

All relevant data are within the paper and its Supporting Information.
