# Supplementary material for: Boundaries potentiate polycomb response element-mediated silencing
Source: BMC Biol. 2021 Jun 2;19:113. doi: 10.1186/s12915-021-01047-8 (PMC8170967; doi:10.1186/s12915-021-01047-8)
Supplement: Supplementary file 1 — Additional file 1: Table S1. Eye phenotypes of hemizygote transgene flies with insertion of white marker gene without enhancer at selected attP lines. Figure S1. X-ChIP for the “Su” transgene with Ph antibody and X-ChIP for the “Su-bxd” and “Su” transgenes with Su(Hw) antibody. Table S2. Primers used for X-ChIP-qPCR analysis to test PcG/TrxG, DNA-binding proteins recruitment to transgenes. Table S3. Primers used for X-ChIP-qPCR analysis to test histone H3 binding to transgenes. Figure S2. Antibody specificity test. [file 12915_2021_1047_MOESM1_ESM.docx]

**Erokhin et al** **Supplementary Information - Additional file 1**

**This file contains:**

**1 –** **Table S1.** Eye phenotypes of heterozygote transgene flies with insertion of *white* marker gene without enhancer at selected attP lines.

**2 –** **Figure S1.** X-ChIP for the “Su” transgene with Ph antibody and X-ChIP for the “Su-bxd” and “Su” transgenes with Su(Hw) antibody.

**3 – Table S2.** Primers used for X-ChIP-qPCR analysis to test PcG/TrxG, DNA-binding proteins recruitment to transgenes.

**4 –** **Table S3.** Primers used for X-ChIP-qPCR analysis to test histone H3 binding to transgenes.

**5 – Figure S2.** Antibody specificity test.

**Table S1.** **Eye phenotypes of hemizygote transgene files with insertion of *white* marker gene at selected attP lines** (according to Bischof J, Maeda RK, Hediger M, Karch F, Basler K: **An optimized transgenesis system for Drosophila using germ-line-specific phiC31 integrases**. *Proceedings of the National Academy of Sciences of the United States of America* 2007, **104**(9):3312-3317.; *white* gene used without eye specific enhancer).

| Line name | Stock number | chromosome | White phenotype |
| --- | --- | --- | --- |
| ZH-attP-22A | BDSC #24481 | 2L | Light orange |
| ZH-attP-51C | BDSC #24482 | 2R | Light orange |
| ZH-attP-58A | BDSC #24484 | 2R | Light orange |
| ZH-attP-68E | BDSC #24485 | 3L | Light orange |
| ZH-attP-96E | BDSC #24487 | 3R | Orange |

**Figure S1** X-ChIP for the “Su” transgene with Ph antibody and X-ChIP for the “Su-bxd” and “Su” transgenes with Su(Hw) antibody.

**
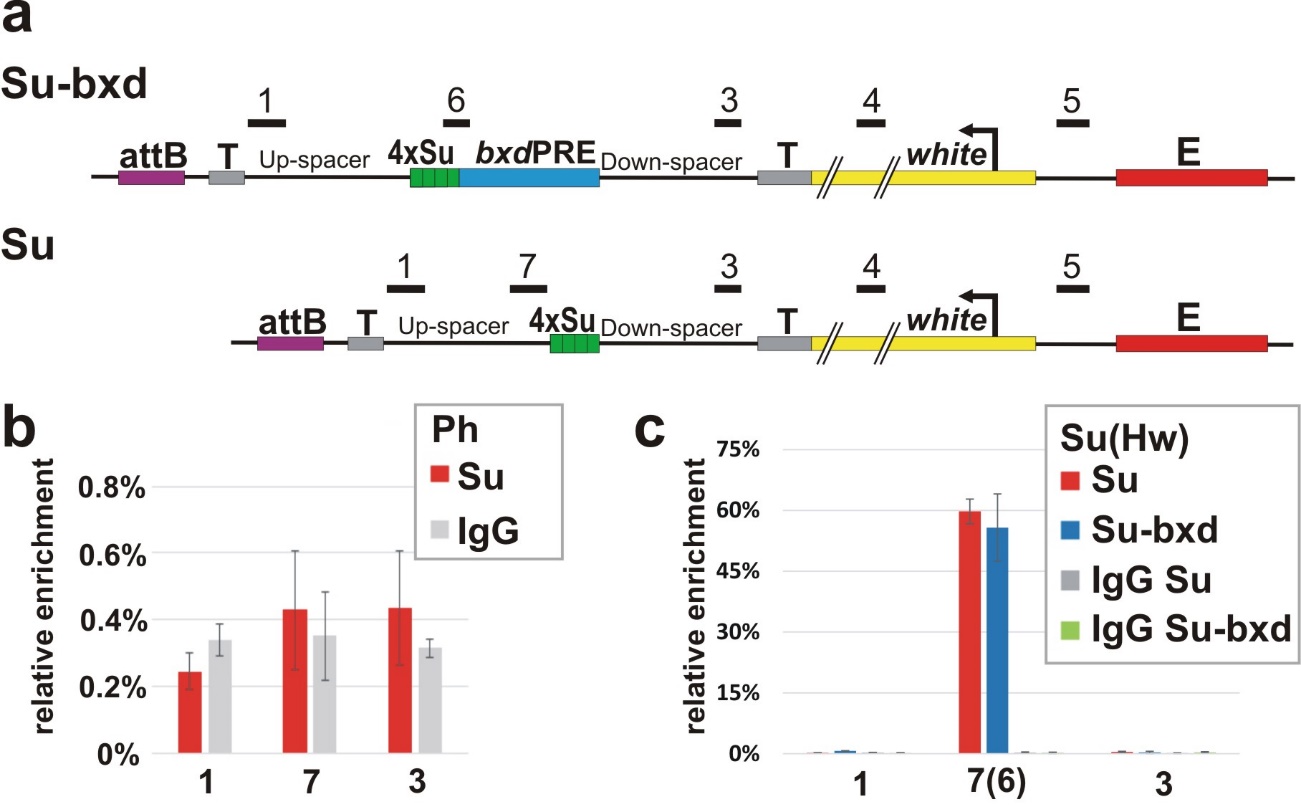
**

(**a**) Maps of the “Su-bxd” and “Su” transgene constructs with the numbers above the constructs (1, 3, 4, 5, 6, 7) indicating regions amplified by qPCR in X-ChIP experiments. The X- ChIPs were performed with chromatin isolated from heads of adult flies homozygous for the “Su-bxd” or “Su” transgenes at 96E insertion site. The X-ChIPs were performed with specific antibodies or with IgG. The specific antibodies: (**b**) PH, (**c**) Su(Hw). (**b**) The ordinate shows the percentage of target sequences in the immunoprecipitated material relative to the input DNA and normalized to *bxd*PRE-Genome. (**c**) The ordinate shows the percentage of target sequences in the immunoprecipitated material relative to the input DNA normalized to 62D Su(Hw) binding genome region. The transgene specific regions and negative genome control (ras - coding part of Ras64B gene) are indicated on the abscissa.

**Table S2.** Primers used for X-ChIP-qPCR analysis to test PcG/TrxG, DNA-binding proteins recruitment to transgenes.

| **Primer name** | **Sequence** |
| --- | --- |
| **bxd, Su-bxd, Su** constructs identical primers | |
| 1-forward (eGFP) | 5’-GTCCATGCCGAGAGTGATCC-3’ |
| 1-reverse (eGFP) | 5’-CCGACCACTACCAGCAGAAC-3’ |
| 3-forward (Cherry) | 5’-GCCACTACGACGCTGAGGTCAA-3’ |
| 3-reverse (Cherry) | 5’-CGTTGTGGGAGGTGATGTCC-3’ |
| 4-forward (*white*) | 5’-GTGGGCTCATCGCAGATCA-3’ |
| 4-reverse (*white*) | 5’-GCAAATGTCAGCACACGATCAT-3’ |
| 5-forward (*white* promoter) | 5’- tggacagagaaggaggcaaaca -3’ |
| 5-reverse (*white* promoter) | 5’- gcactggatatcattgaacttatctg -3’ |
| **bxd** construct | |
| 2-forward (from eGFP) | 5’-AGCTCCTCGCCCTTGCTCACCAT-3’ |
| 2-reverse (from bxdPRE) | 5’-CCATAATGGCTGCGCCGTAAAG-3’ |
| **Su-bxd** construct | |
| 6-forward (from 4xSu-polylinker) | 5’-TTTTGAGATCCACTAGTGAGGCA-3’ |
| 6-reverse = 2-reverse (from bxdPRE) | 5’-CCATAATGGCTGCGCCGTAAAG-3’ |
| **Su** construct | |
| 7-forward (from eGFP) | 5’-CGGTGGTGCAGATGAACTTC-3’ |
| 7-reverse (from eGFP) | 5’-AGCAAGGGCGAGGAGCTGTT-3’ |
| **en, Su-en** constructs | |
| en-forward (from enPRE) | 5’-AGATGGCATGTGGCTCTCCC-3’ |
| en-reverse (from Cherry) | 5-TTCAGCTTGGCGGTCTGGGT-3’ |
| Control regions | |
| bxdPRE-*Genome*-forward | 5’-AAGAGCAAGGCGAAAGAGAGC-3’ |
| bxdPRE-*Genome*-reverse | 5’-CGTTTTAAGTGCGACTGAGATGG-3’ |
| Ras64B-forward | 5'-gagggattcctgctcgtcttcg-3' |
| Ras64B-reverse | 5'-gtcgcacttgttacccaccatc-3' |
| 62D-forward | 5'-TGATACCAGGCGAACAGAAATC-3' |
| 62D-reverse | 5'-TTTGGGCTTGGTGAGAACAG-3' |

**Table S3.** Primers used for X-ChIP-qPCR analysis to test histone H3 binding to transgenes.

| **Primer name** | **Sequence** |
| --- | --- |
| Constructs primers | |
| 1n-direct | 5'-TCGCCCTCGAACTTCACCTC-3' |
| 1n-reverse | 5'-ACCCCGACCACATGAAGCAG-3' |
| 2n-direct | 5'-AAGCACTGCACGCCGTAGGTC-3' |
| 2n-reverse | 5'-CCTACGGCAAGCTGACCCTGAA-3' |
| 3n-direct | 5'-TCGCCGGACACGCTGAACTT-3' |
| 3n-reverse | 5'-CAAGGGCGAGGAGCTGTTCA-3' |
| 4n-direct | 5'-TGAGCAAGGGCGAGGAGGAT-3' |
| 4n-reverse | 5'-TTCAGCTTGGCGGTCTGGGT-3' |
| 5n-direct | 5'-CTTCGCCTGGGACATCCTGT-3' |
| 5n-reverse | 5'-CGGGGAAGGACAGCTTCAAG-3' |
| 6n-direct | 5'-GGGAGCGCGTGATGAACTTCGA-3' |
| 6n-reverse | 5'-TCTTCTGCATTACGGGGCCGTC-3' |
| Control point primers | |
| Ras64B-forward | 5'-gagggattcctgctcgtcttcg-3' |
| Ras64B-reverse | 5'-gtcgcacttgttacccaccatc-3' |

**Figure S2.** Antibody specificity test.

**
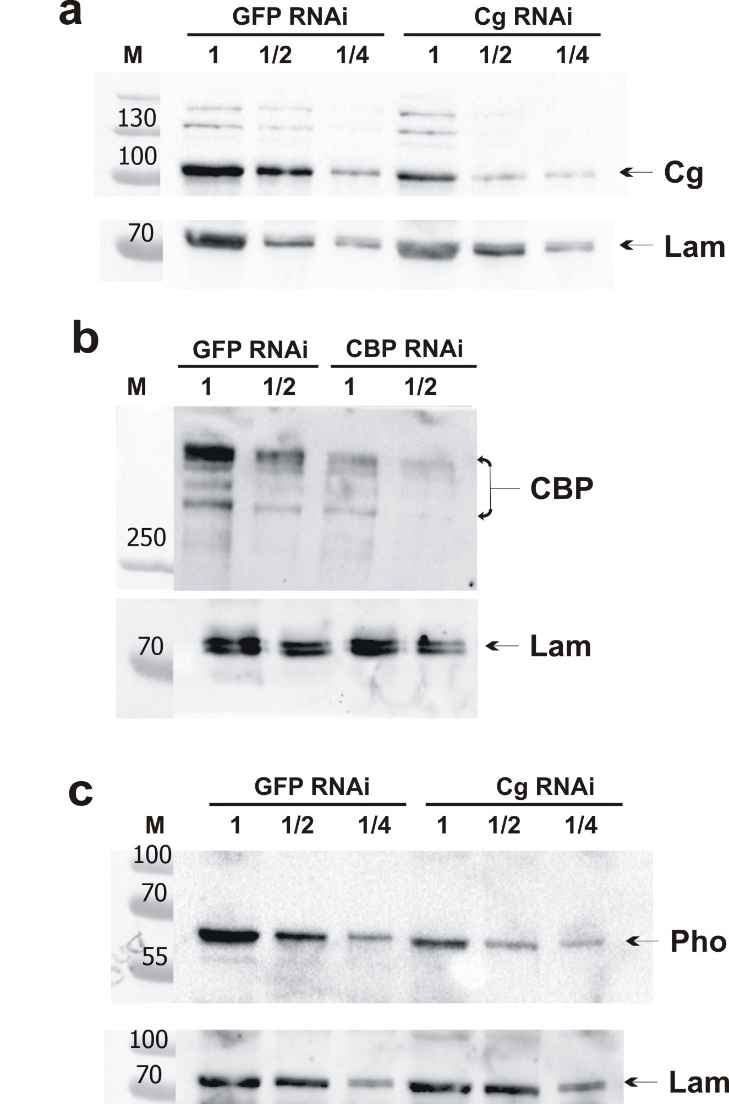
**

Knockdown of (**a**) Cg, (**b**) CBP and (**c**) Pho in S2 Drosophila cells was performed as described previously (Mazina MY, Kovalenko EV, Derevyanko PK, Nikolenko JV, Krasnov AN, Vorobyeva NE: **One signal stimulates different transcriptional activation mechanisms**. *Biochimica et biophysica acta Gene regulatory mechanisms* 2018, **1861**(2):178-189). S2 cells were treated sequentially with dsRNA for two and three days. Utilized dsRNAs corresponded to the following fragments of the transcripts: 967-1356 b. and 2067-2553 of cg-RL; 2283-2789 b. and 7168-7683 b. of nej-RB; 494-954 b. and 1476-1889 b. of pho-RA.
