# Supplementary material for: Boundaries potentiate polycomb response element-mediated silencing
Source: BMC Biol. 2021 Jun 2;19:113. doi: 10.1186/s12915-021-01047-8 (PMC8170967; doi:10.1186/s12915-021-01047-8)

This file contains:

**the analysis of homologue chromosomes contact frequencies at the 22A, 51C, 58A, 68E and 96E attP insertion sites**: 22A (Bloomington Drosophila Stock Center (BDSC) #24481), 51C (BDSC #24482), 58A (BDSC #24484), 68E (BDSC #24485), 96E (BDSC #24487).

Description:

- For the analysis Haplotype-resolved Hi-C contacts from accessions GSM3428928 and GSM3428929 were used (AlHaj Abed J, Erceg J, Goloborodko A, Nguyen SC et al. Highly structured homolog pairing reflects functional organization of the Drosophila genome. *Nat Commun* 2019 Oct 3;10(1):4485. PMID: [31582763](https://www.ncbi.nlm.nih.gov/pubmed/31582763)).
- Contact maps of 2 Mb regions centered on the insertion sites of transgenes (indicated by dashed lines). cis-Mat or cis-Pat — cis interactions occurring within maternal or paternal chromosome, respectively. thom — trans interactions between homologous chromosomes. O/E – observed over expected on that genomic separation contact frequency. Bottom left map (thom/cis) shows depletion of contacts near 58A attP insertion site.
- Processed Hi-C contact pairs with high mapping quality (MAPQ>30) on both sides were aggregated in 20kb genomic bins to produce Hi-C contact matrices using the *cooler* v0.8.10 package (<https://doi.org/10.1093/bioinformatics/btz540>). Matrices were iteratively corrected (<http://dx.doi.org/10.1038/nmeth.2148>) after low-quality bin filtering using cooler *balance* function with default parameters. Contact frequency as a function of genomic separation was calculated by averaging normalised contact frequency along diagonals of contact matrices using *cooltools* v0.3.2 package (<https://github.com/open2c/cooltools>).

1. 22A


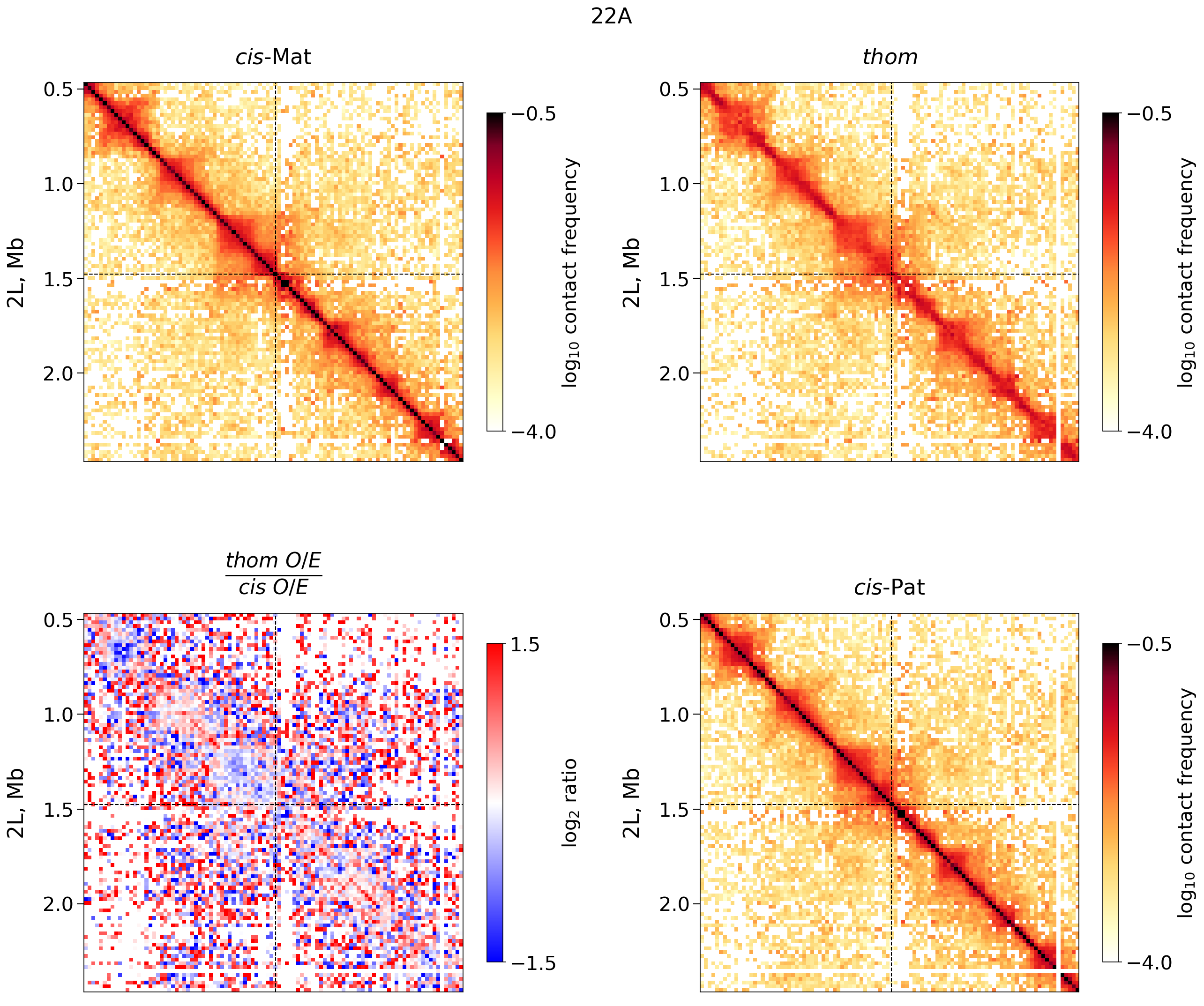


1. 51C


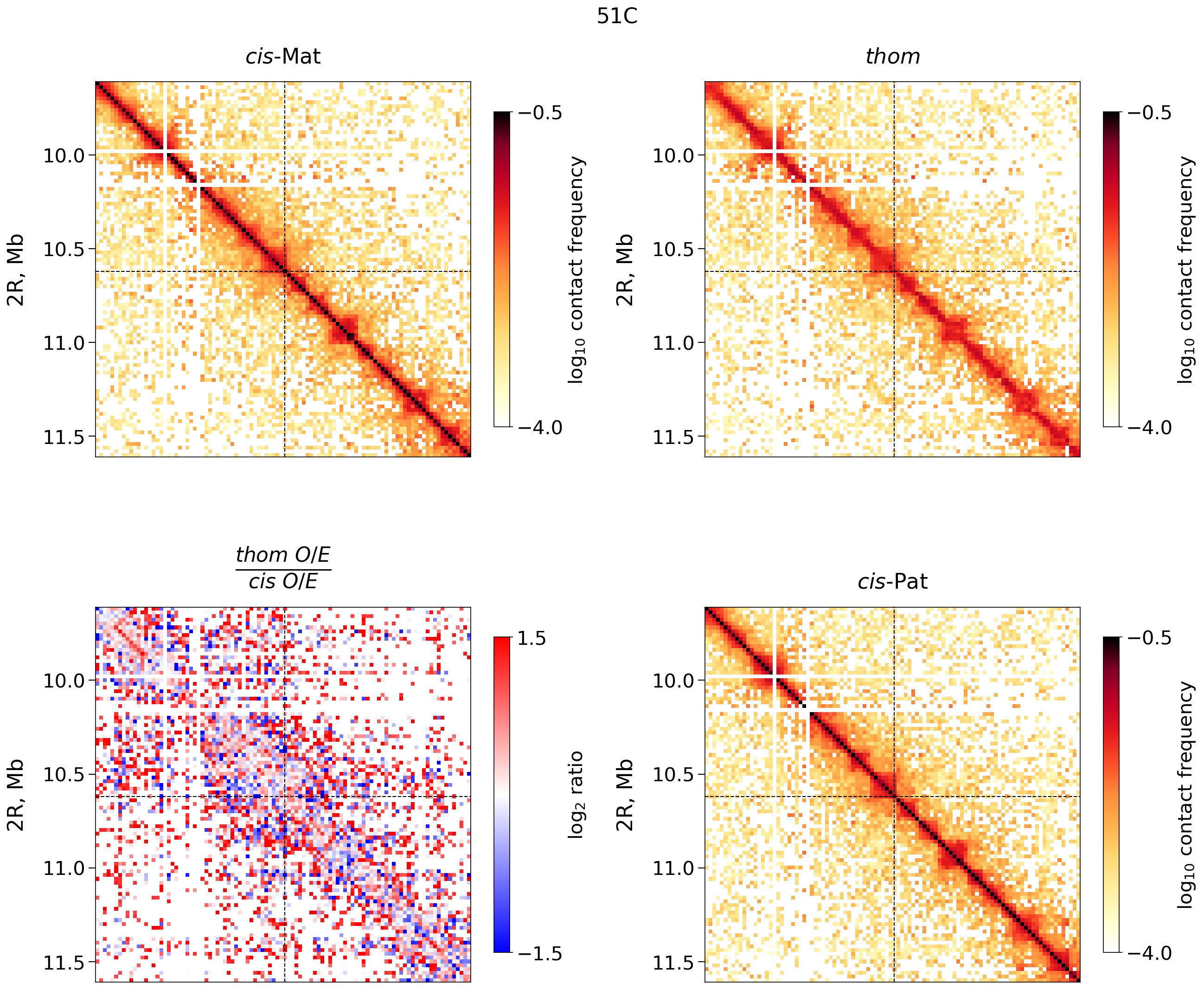


1. 58A


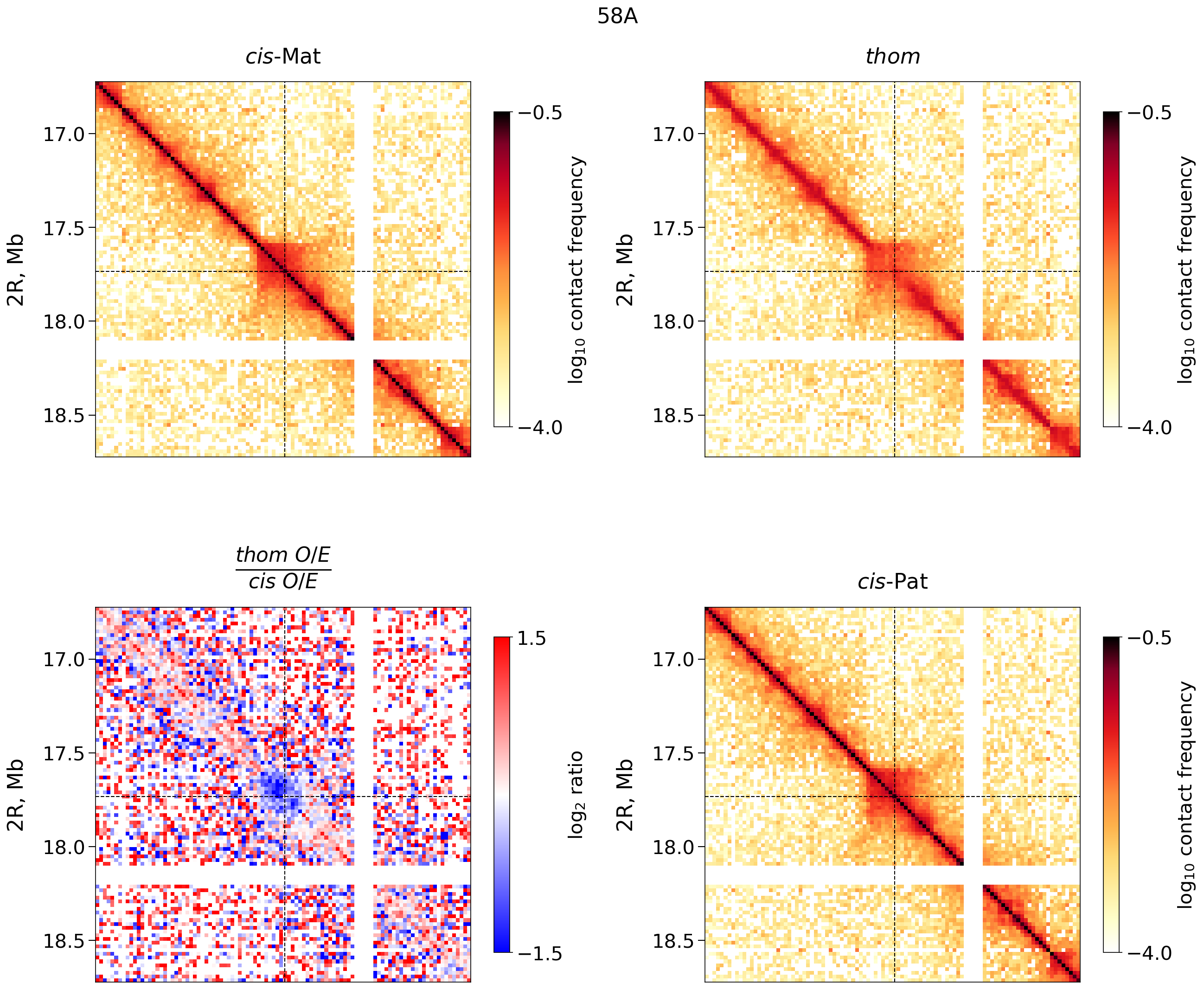


1. 68E


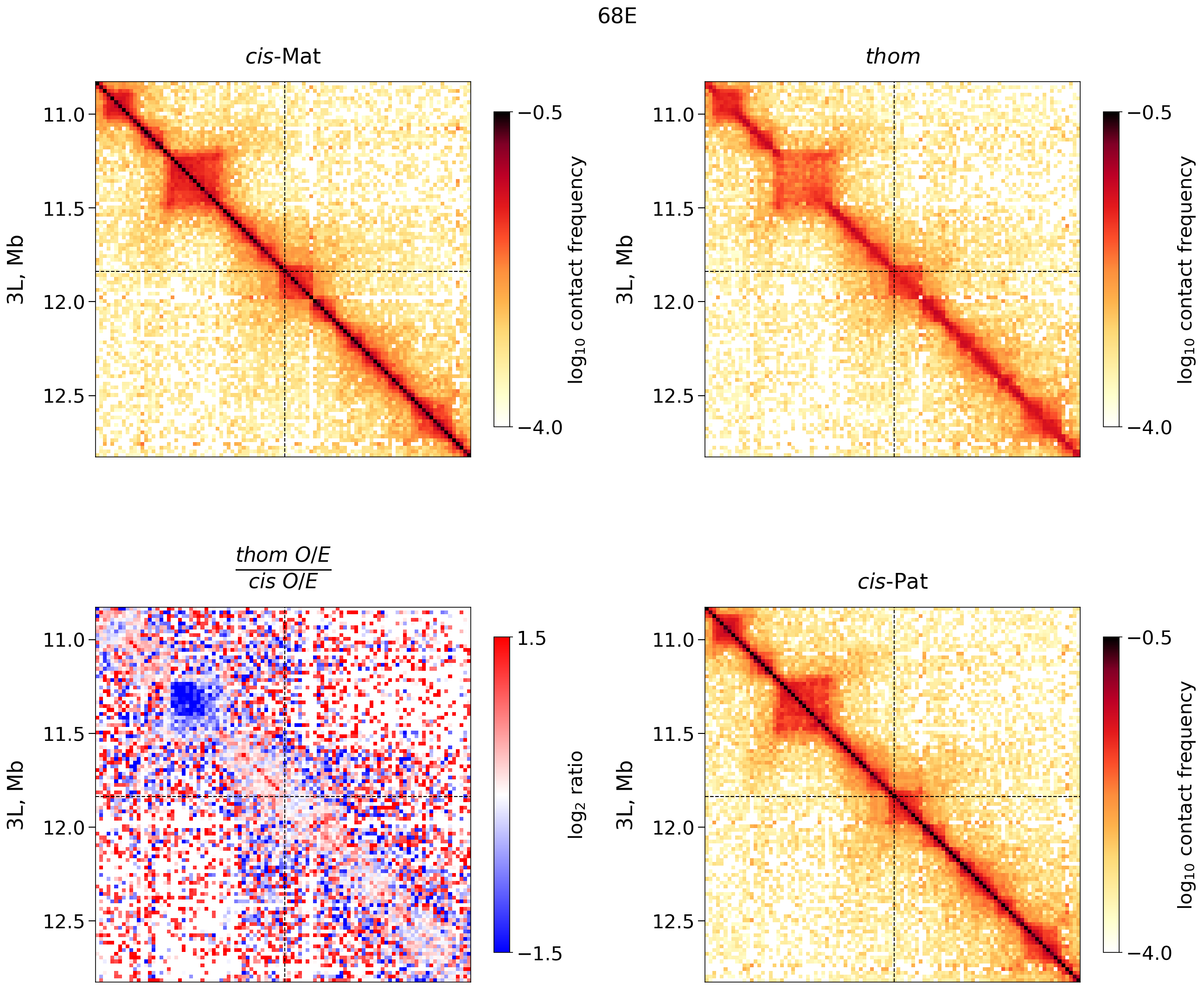


1. 96E


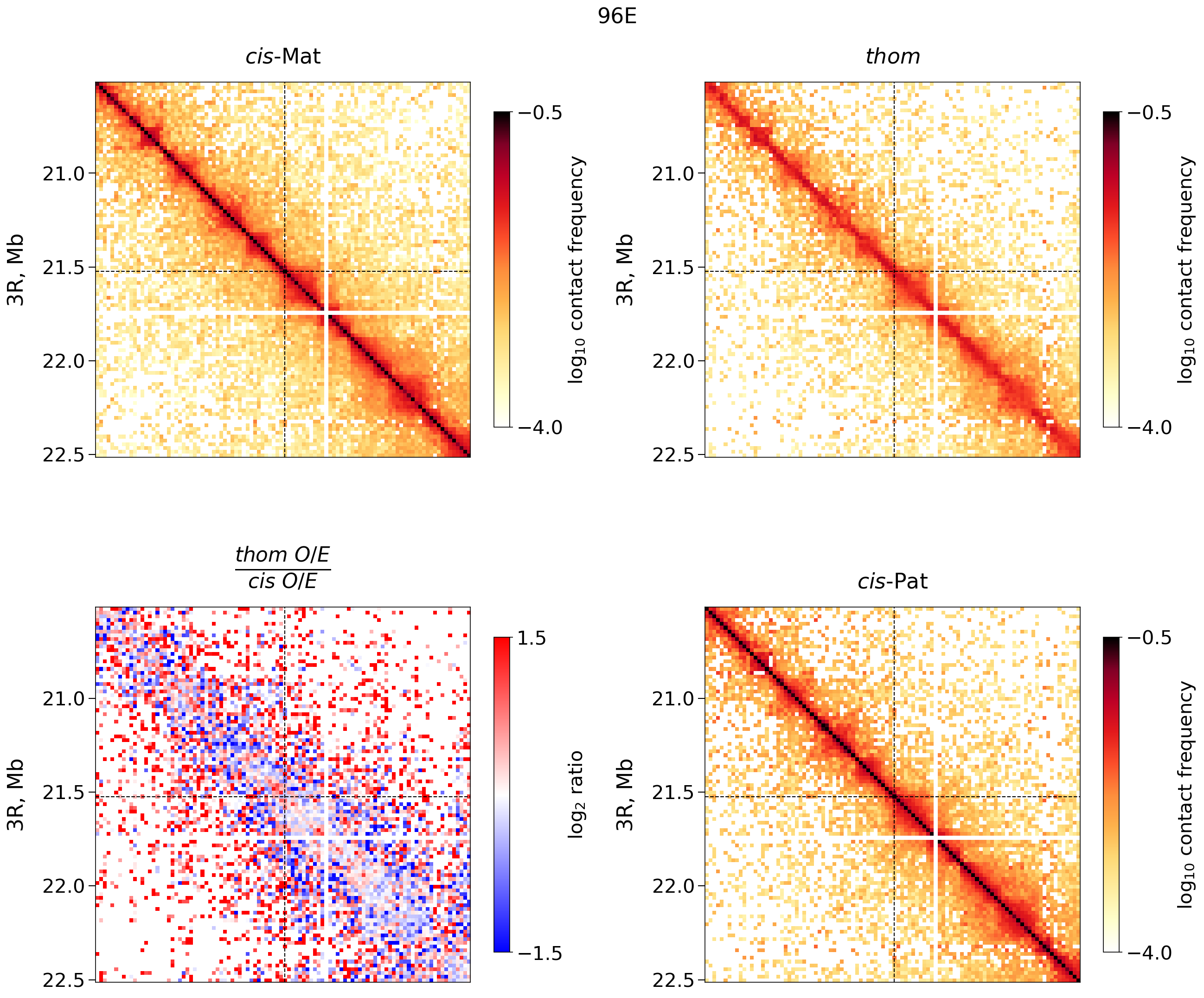

Supplement: Supplementary file 2 — Additional file 2. This file contains the analysis of homologue chromosomes contact frequencies at the 22A, 51C, 58A, 68E and 96E attP insertion sites. [file 12915_2021_1047_MOESM2_ESM.docx]
