# Supplementary material for: Boundaries potentiate polycomb response element-mediated silencing
Source: BMC Biol. 2021 Jun 2;19:113. doi: 10.1186/s12915-021-01047-8 (PMC8170967; doi:10.1186/s12915-021-01047-8)

Erokhin et al Supplementary Information - Additional file 2

This file contains the ChIP-seq data for PcG and boundary proteins binding to 22A, 51C, 58A, 68E and 96E genome regions.

1) **22A**
2) **51C**
3) **58A**
4) **68E**
5) **96E**

The boundary data from Ramirez F, Bhardwaj V, Arrigoni L, Lam KC, Gruning BA, Villaveces J, Habermann B, Akhtar A, Manke T: **High-resolution TADs reveal DNA sequences underlying genome organization in flies**. *Nature communications* 2018, **9**(1):189.

The PcG, H3K27me3 data from Brown JL, Sun MA, Kassis JA: **Global changes of H3K27me3 domains and Polycomb group protein distribution in the absence of recruiters Spps or Pho**. *Proceedings of the National Academy of Sciences of the United States of America* 2018, **115**(8):E1839-E1848.

1) 22A

Boundary proteins

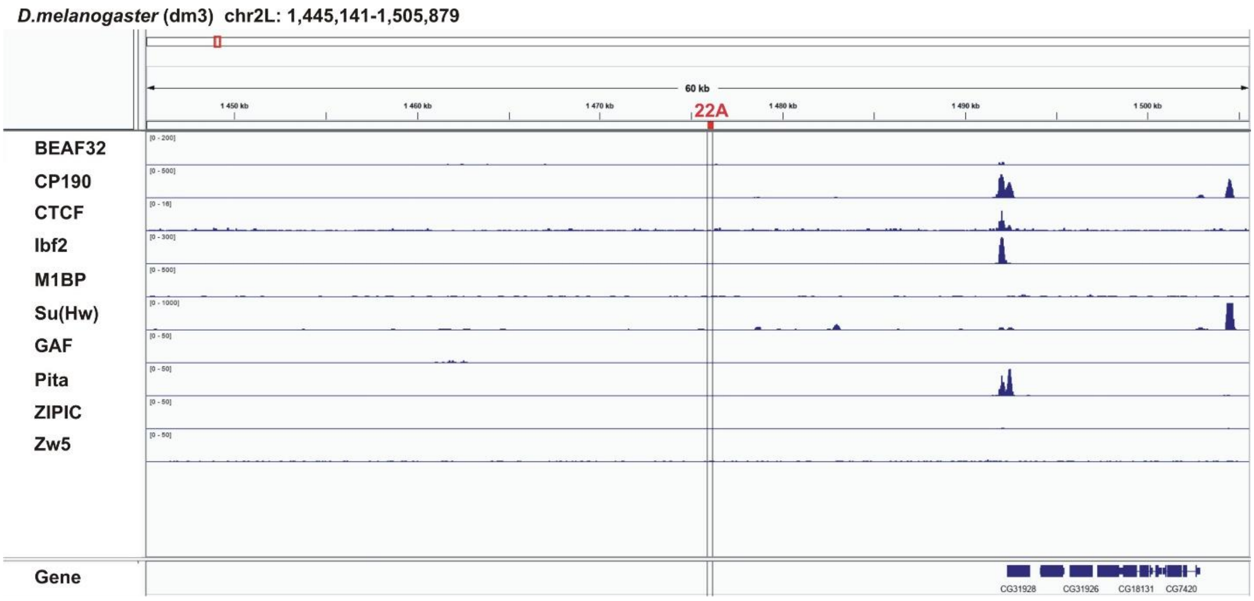

PcG proteins and H3K27me3 histone modification

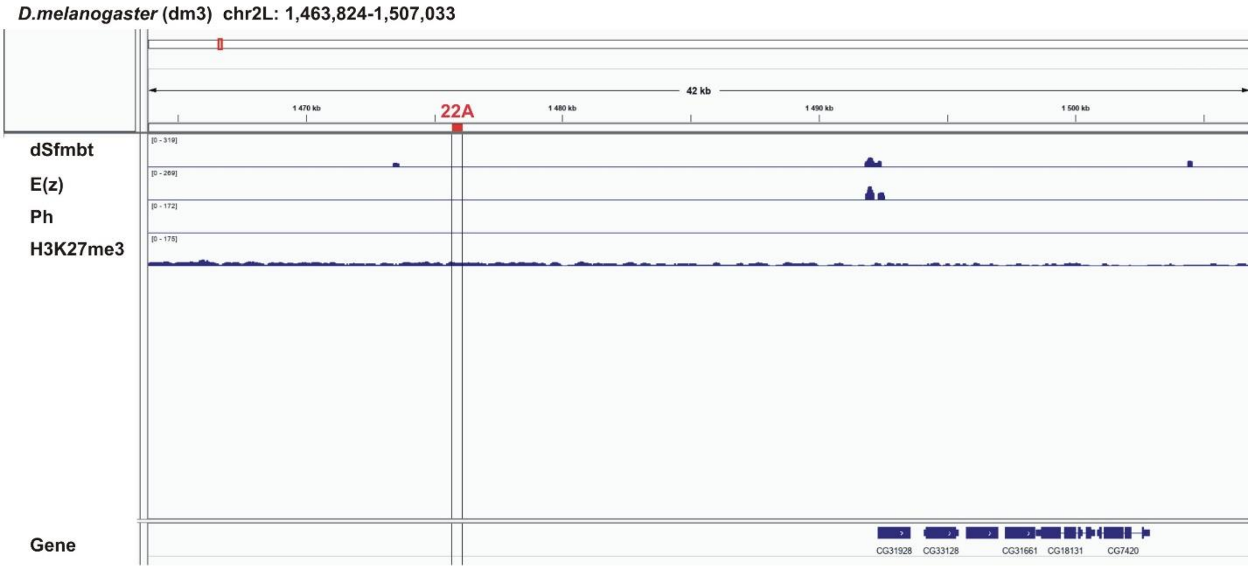

2) 51C

Boundary proteins

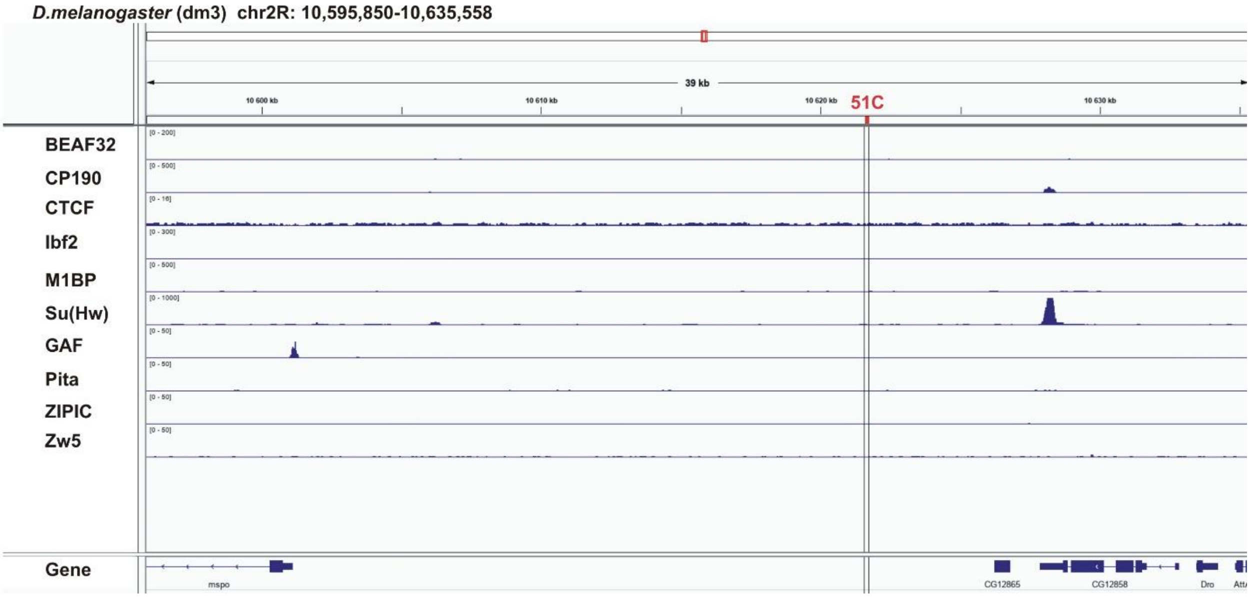

PcG proteins and H3K27me3 histone modification

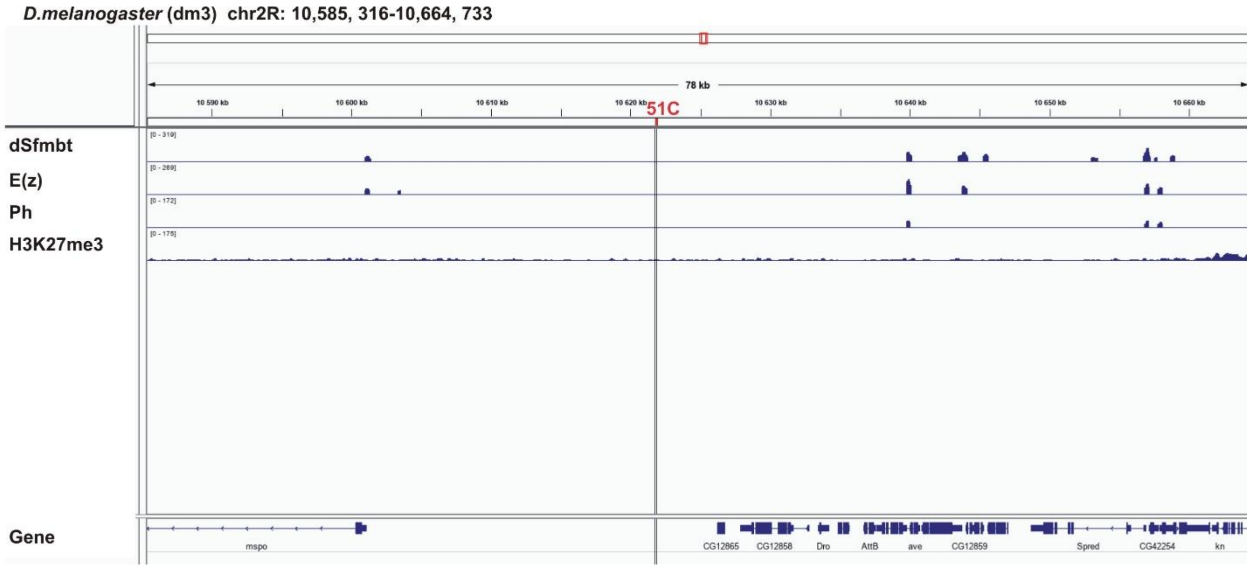

3) 58A

Boundary proteins

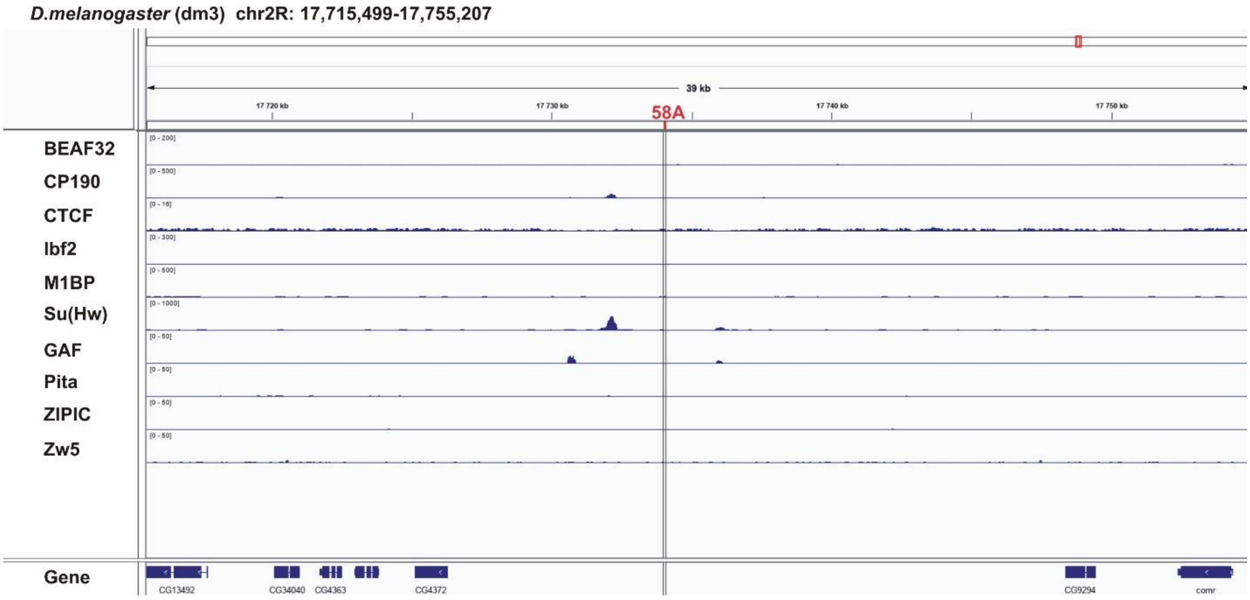

PcG proteins and H3K27me3 histone modification

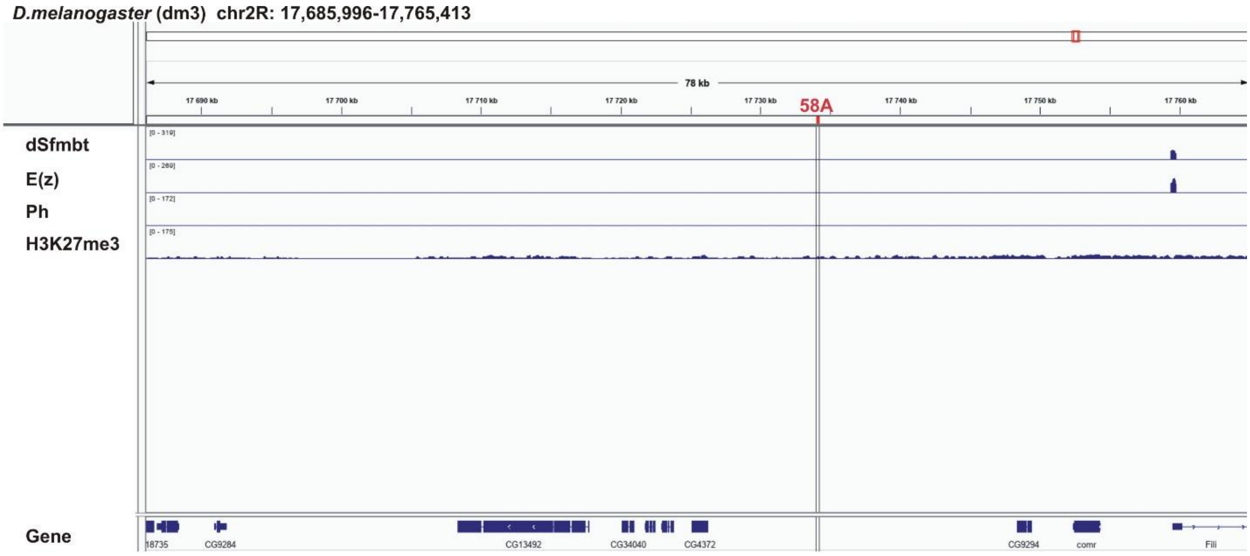

4) 68E

Boundary proteins

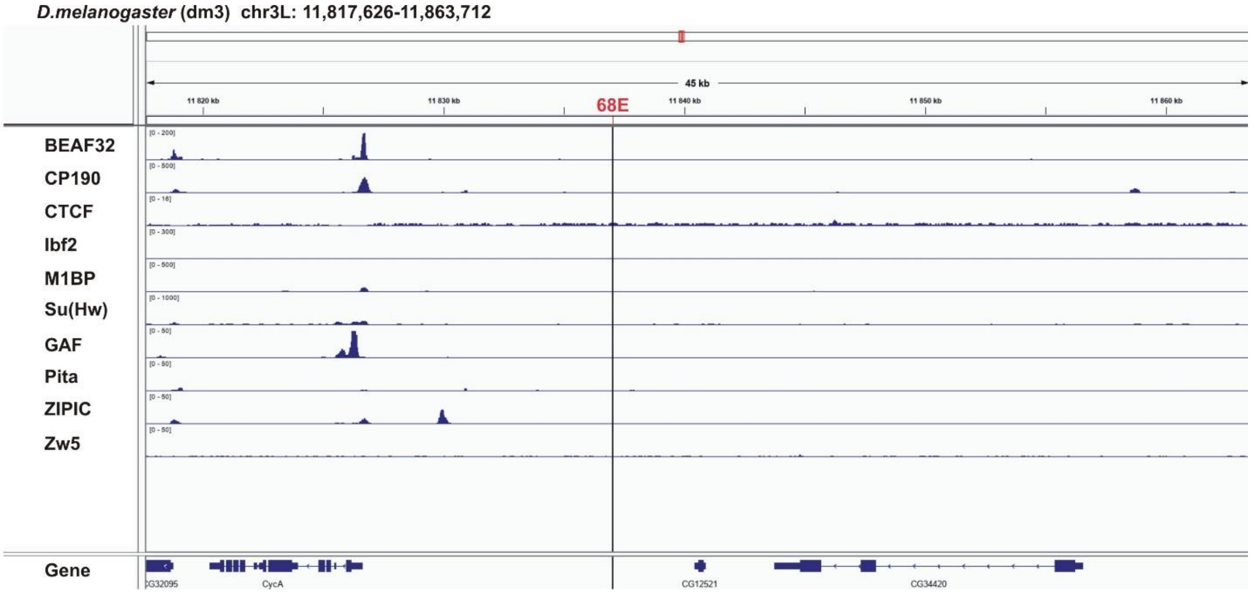

PcG proteins and H3K27me3 histone modification

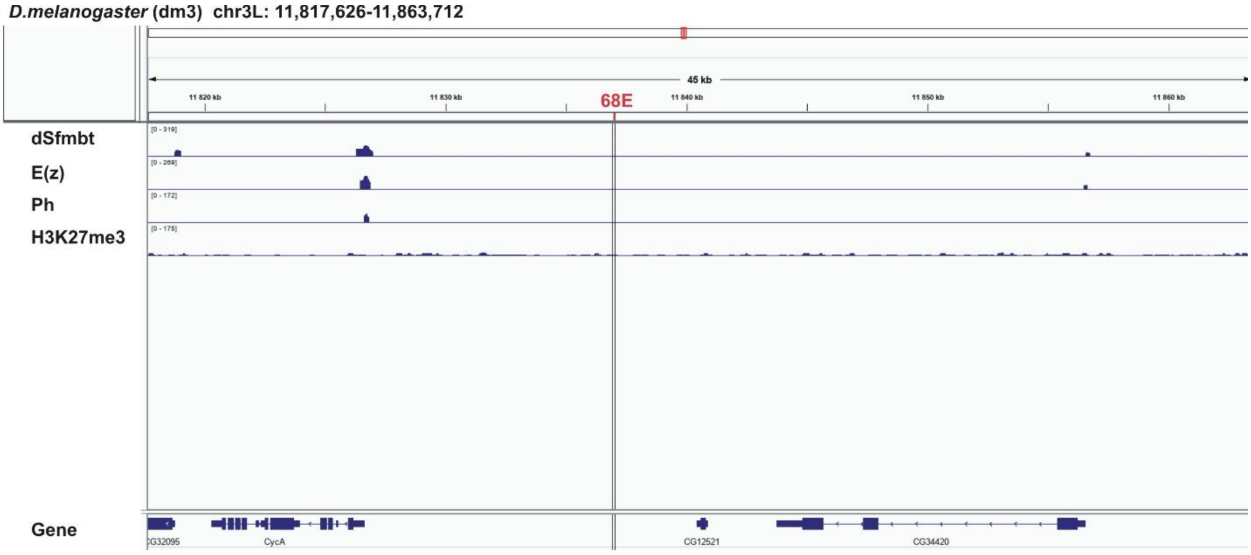

5) 96E

Boundary proteins

D.melanogaster (dm3) chr3R: 21,497,025-21,549,423

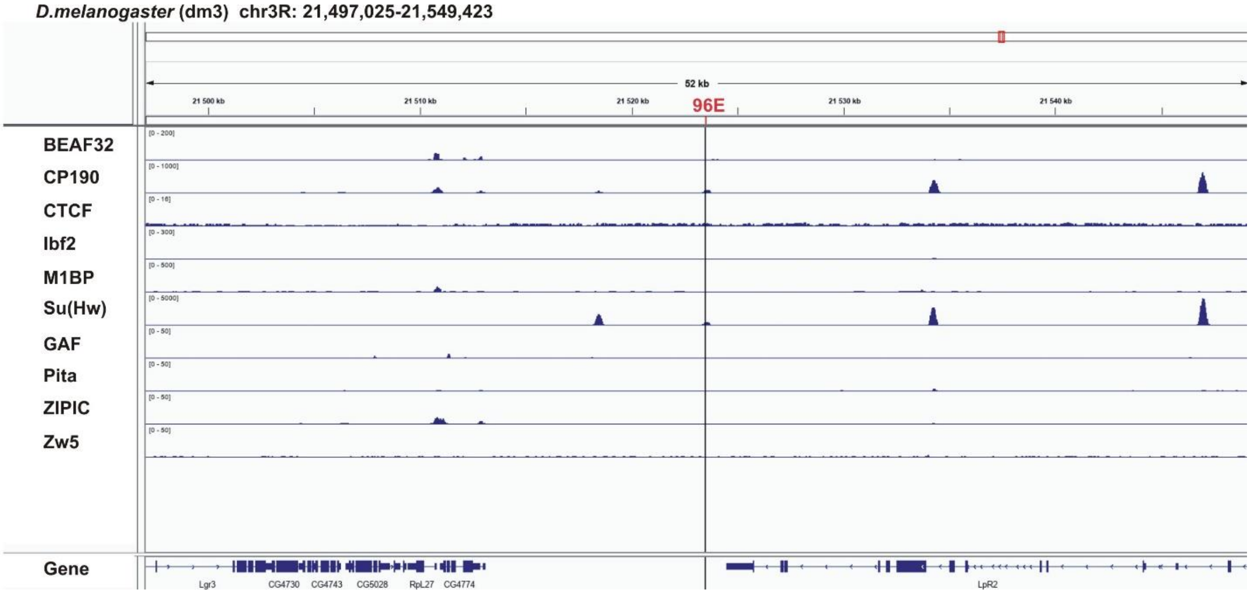

Boundary proteins (higher resolution)

D.melanogaster (dm3) chr3R: 21,510,408-21,536,606

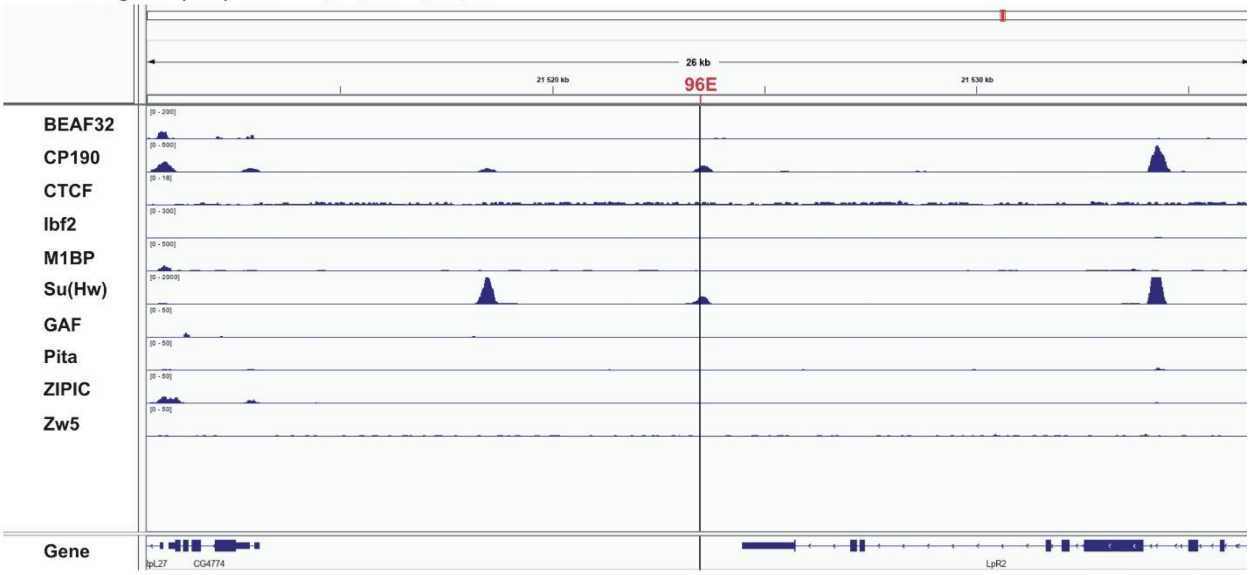

PcG proteins and H3K27me3 histone modification

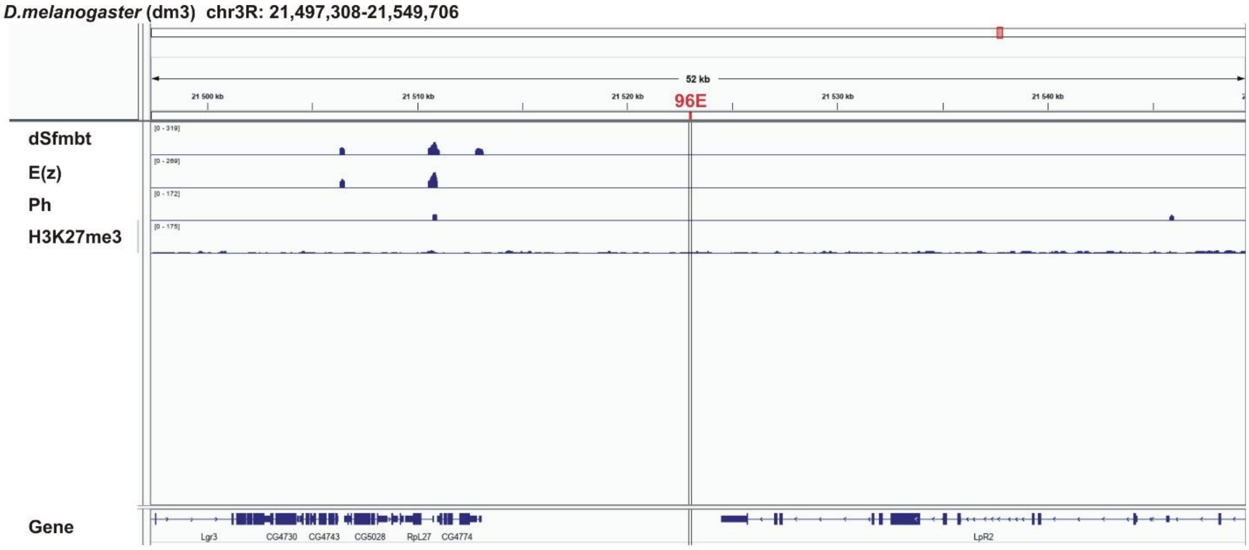

Supplement: Supplementary file 3 — Additional file 3. This file contains the ChIP-seq data for PcG and boundary proteins binding to 22A, 51C, 58A, 68E and 96E genome regions. [file 12915_2021_1047_MOESM3_ESM.pdf]
