# Supplementary material for: Boundaries potentiate polycomb response element-mediated silencing
Source: BMC Biol. 2021 Jun 2;19:113. doi: 10.1186/s12915-021-01047-8 (PMC8170967; doi:10.1186/s12915-021-01047-8)
Supplement: Supplementary file 4 — Additional file 4 This file contains: (1) the DNA sequences of the construct elements. (2) the details of Drosophila genetic crosses. [file 12915_2021_1047_MOESM4_ESM.docx]

**Erokhin et al Supplementary Information - Additional file 3**

This file contains: **(1)** the DNA sequences of the construct elements. **(2)** the details of Drosophila genetic crosses.

**(1)**

**The sequences of the functional construct elements:**

**attB**:

gtcgacatgcccgccgtgaccgtcgagaacccgctgacgctgccccgcgtatccgcacccgccgacgccgtcgcacgtcccgtgctcaccgtgaccaccgcgcccagcggtttcgagggcgagggcttcccggtgcgccgcgcgttcgccgggatcaactaccgccacctcgacccgttcatcatgatggaccagatgggtgaggtggagtacgcgcccggggagcccaagggcacgccctggcacccgcaccgcggcttcgagaccgtgacctacatcgtcgac

**SV40 terminator:**

aacttgtttattgcagcttataatggttacaaataaagcaatagcatcacaaatttcacaaataaagcatttttttcactgcattctagttgtggtttgtccaaactcatcaatgtatcttatcatgtctg

**eGFP coding sequence (inserted in reverse orientation relative to 5’construct end):**

atggtgagcaagggcgaggagctgttcaccggggtggtgcccatcctggtcgagctggacggcgacgtaaacggccacaagttcagcgtgtccggcgagggcgagggcgatgccacctacggcaagctgaccctgaagttcatctgcaccaccggcaagctgcccgtgccctggcccaccctcgtgaccaccctgacctacggcgtgcagtgcttcagccgctaccccgaccacatgaagcagcacgacttcttcaagtccgccatgcccgaaggctacgtccaggagcgcaccatcttcttcaaggacgacggcaactacaagacccgcgccgaggtgaagttcgagggcgacaccctggtgaaccgcatcgagctgaagggcatcgacttcaaggaggacggcaacatcctggggcacaagctggagtacaactacaacagccacaacgtctatatcatggccgacaagcagaagaacggcatcaaggtgaacttcaagatccgccacaacatcgaggacggcagcgtgcagctcgccgaccactaccagcagaacacccccatcggcgacggccccgtgctgctgcccgacaaccactacctgagcacccagtccgccctgagcaaagaccccaacgagaagcgcgatcacatggtcctgctggagttcgtgaccgccgccgggatcactctcggcatggacgagctgtacaagatcaag

**4xSu** (Green – the Su(Hw) binding site from the gypsy retrotransposon, binding site core – bold).

ggatctcaaaaaataag**tgctgcatactt**tttaggatctcaaaaaataag**tgctgcatactt**ttaggatctcaaaaaataag**tgctgcatactt**tttaggatctcaaaaaataag**tgctgcatactt**tttaggatcatc

**4xCTCF** (Green – CTCF-binding site).

gctgcagcgccacctggccttggagatcctgcagcgccacctggccttggagatcctgcagcgccacctggccttggagatctccaaggccaggtggcgctgcagccccgggctgcag

**5xPita** (Green – Pita-binding site).

gatctttagccaagacgcgaacccgaatccgaaactttagccaagacgcgaacccgaatccgaaactttagccaagacgcgaacccgaatccgaaacttagccaagacgcgaacccgaatccgaaactttagccaagacgcgaacccgaatccgaagatctaatatc

**bxdPRE 656bp:**

gaggcagcgactgcgccgcgactgcggagagagggagagatacggttagccttctcgctcggatcgctctcgctttacggcgcagccattatggtgcgcgtagtcttatctgtatctcgctcttacgcacgtcagacttggaatagccctctctctttttgagttatcggcactttggttctgccgttatggcttccgtgtgaagggggcgtggcctagagagcagtagcatttcgcactggggttttatggctcattttcggattttgagtgcgttcttccgccgcttcttcttctttttcgggcttgtattcgtgttttgtattttctagggttccttctttgacatttgccgtcgcttgtttggataattacttggattgttattagattgttgcaacatctataaaagggccgtaaaaaatatttttatttcgctttaagtgatttttagtggccttgcggtgacaaattgctccggcaacagaagattatggaccttatcaccgcacttatattgttgttcttaactttggatgcactcataaaaaaacttggtcatgtataaaggctcatgaaagtctcgttttaaatattaatttagggacaataataaaacaagtttttgttgaaaaagatatttttatcttgggttg

**enPRE 188 bp:**

gagatggcatgtggctctccccctcatggaaaggcagccattttcctggcctactcgcagagggagtgaacagtgccgctatatgacccccactcggtaacgccccgtgagagagggagagcttgcgcctgcgcagttcgctggggacacagttgtcaaccggctgacagctccagcatgcgcataat

**Cherry coding sequence:**

atggtgagcaagggcgaggaggataacatggccatcatcaaggagttcatgcgcttcaaggtgcacatggagggctccgtgaacggccacgagttcgagatcgagggcgagggcgagggccgcccctacgagggcacccagaccgccaagctgaaggtgaccaagggtggccccctgcccttcgcctgggacatcctgtcccctcagttcatgtacggctccaaggcctacgtgaagcaccccgccgacatccccgactacttgaagctgtccttccccgagggcttcaagtgggagcgcgtgatgaacttcgaggacggcggcgtggtgaccgtgacccaggactcctccctgcaggacggcgagttcatctacaaggtgaagctgcgcggcaccaacttcccctccgacggccccgtaatgcagaagaagaccatgggctgggaggcctcctccgagcggatgtaccccgaggacggcgccctgaagggcgagatcaagcagaggctgaagctgaaggacggcggccactacgacgctgaggtcaagaccacctacaaggccaagaagcccgtgcagctgcccggcgcctacaacgtcaacatcaagttggacatcacctcccacaacgaggactacaccatcgtggaacagtacgaacgcgccgagggccgccactccaccggcggcatggacgagctgtacaagtaa

**2087 bp lacZ containing fragment:**

cgttatcgctatgacggaacaggtattcgctggtcacttcgatggtttgcccggataaacggaactggaaaaactgctgctggtgttttgcttccgtcagcgctggatgcggcgtgcggtcggcaaagaccagaccgttcatacagaactggcgatcgttcggcgtatcgccaaaatcaccgccgtaagccgaccacgggttgccgttttcatcatatttaatcagcgactgatccacccagtcccagacgaagccgccctgtaaacggggatactgacgaaacgcctgccagtatttagcgaaaccgccaagactgttacccatcgcgtgggcgtattcgcaaaggatcagcgggcgcgtctctccaggtagcgaaagccattttttgatggaccatttcggcacagccgggaagggctggtcttcatccacgcgcgcgtacatcgggcaaataatatcggtggccgtggtgtcggctccgccgccttcatactgcaccgggcgggaaggatcgacagatttgatccagcgatacagcgcgtcgtgattagcgccgtggcctgattcattccccagcgaccagatgatcacactcgggtgattacgatcgcgctgcaccattcgcgttacgcgttcgctcatcgccggtagccagcgcggatcatcggtcagacgattcattggcaccatgccgtgggtttcaatattggcttcatccaccacatacaggccgtagcggtcgcacagcgtgtaccacagcggatggttcggataatgcgaacagcgcacggcgttaaagttgttctgcttcatcagcaggatatcctgcaccatcgtctgctcatccatgacctgaccatgcagaggatgatgctcgtgacggttaacgcctcgaatcagcaacggcttgccgttcagcagcagcagaccattttcaatccgcacctcgcggaaaccgacatcgcaggcttctgcttcaatcagcgtgccgtcggcggtgtgcagttcaaccaccgcacgatagagattcgggatttcggcgctccacagtttcgggttttcgacgttcagacgtagtgtgacgcgatcggcataaccaccacgctcatcgataatttcaccgccgaaaggcgcggtgccgctggcgacctgcgtttcaccctgccataaagaaactgttacccgtaggtagtcacgcaactcgccgcacatctgaacttcagcctccagtacagcgcggctgaaatcatcattaaagcgagtggcaacatggaaatcgctgatttgtgtagtcggtttatgcagcaacgagacgtcacggaaaatgccgctcatccgccacatatcctgatcttccagataactgccgtcactccaacgcagcaccatcaccgcgaggcggttttctccggcgcgtaaaaatgcgctcaggtcaaattcagacggcaaacgactgtcctggccgtaaccgacccagcgcccgttgcaccacagatgaaacgccgagttaacgccatcaaaaataattcgcgtctggccttcctgtagccagctttcatcaacattaaatgtgagcgagtaacaacccgtcggattctccgtgggaacaaacggcggattgaccgtaatgggataggttacgttggtgtagatgggcgcatcgtaaccgtgcatctgccagtttgaggggacgacgacagtatcggcctcaggaagatcgcactccagccagctttccggcaccgcttctggtgccggaaaccaggcaaagcgccattcgccattcaggctgcgcaactgttgggaagggcgatcggtgcgggcctcttcgctattacgccagctggcgaaagggggatgtgctgcaaggcgattaagttgggtaacgccagggttttcccagtcacgacgttgtaaaacgacgggatcgcgcttgagcagctccttgctggtgtccagaccaatgcctcccagaccggcaacgaaaatcacgttcttgttggtcaaagtaaacgacatggtgacttcttttttgctttagcaggctctttcgatccccgggggatccactagttctagagcggcc

***yellow* terminator (inserted in reverse orientation relative to 5’construct end):**

agaaaatgtacagttgttgatatatttagaatttatgcatacttacattttttccgctttttccgctcaagaaaattgcgtaaactcttaaccttcaaaaaaaagttgatttattgttattttttgcttaacataactagataaagtattgatttgccacttgctcatacgtcatgtggtttttttaaccgctgttgccctatgttgttgtctttaaatattctttacatca

***white* (*mini-white*) gene without Wari-insulator (inserted in reverse orientation relative to 5’construct end)** (Red – transcription start site)**:**

actgcactggatatcattgaacttatctgatcagttttaaatttacttcgatccaagggtatttgaagtaccaggttctttcgattacctctcactcaaaatgacattccactcaaagtcagcgctgtttgcctccttctctgtccacagaaatatcgccgtctctttcgccgctgcgtccgctatctctttcgccaccgtttgtagcgttacctagcgtcaatgtccgccttcagttgcactttgtcagcggtttcgtgacgaagctccaagcggtttacgccatcaattaaacacaaagtgctgtgccaaaactcctctcgcttcttatttttgtttgttttttgagtgattggggtggtgattggttttgggtgggtaagcaggggaaagtgtgaaaaatcccggcaatgggccaagaggatcaggagctattaattcgcggaggcagcaaacacccatctgccgagcatctgaacaatgtgagtagtacatgtgcatacatcttaagttcacttgatctataggaactgcgattgcaacatcaaattgtctgcggcgtgagaactgcgacccacaaaaatcccaaaccgcaatcgcacaaacaaatagtgacacgaaacagattattctggtagctgtgctcgctatataagacaatttttaagatcatatcatgatcaagacatctaaaggcattcattttcgactacattcttttttacaaaaaatataacaaccagatattttaagctgatcctagatgcacaaaaaataaataaaagtataaacctacttcgtaggatacttcgttttgttcggggttagatgagcataacgcttgtagttgatatttgagatcccctatcattgcagggtgacagcggacgcttcgcagagctgcattaaccagggcttcgggcaggccaaaaactacggcacgctcctgccacccagtccgccggaggactccggttcagggagcggccaactagccgagaacctcacctatgcctggcacaatatggacatctttggggcggtcaatcagccgggctccggatggcggcagctggtcaaccggacacgcggactattctgcaacgagcgacacataccggcgcccaggaaacatttgctcaagaacggtgagtttctattcgcagtcggctgatctgtgtgaaatcttaataaagggtccaattaccaatttgaaactcagtttgcggcgtggcctatccgggcgaacttttggccgtgatgggcagttccggtgccggaaagacgaccctgctgaatgcccttgcctttcgatcgccgcagggcatccaagtatcgccatccgggatgcgactgctcaatggccaacctgtggacgccaaggagatgcaggccaggtgcgcctatgtccagcaggatgacctctttatcggctccctaacggccagggaacacctgattttccaggccatggtgcggatgccacgacatctgacctatcggcagcgagtggcccgcgtggatcaggtgatccaggagctttcgctcagcaaatgtcagcacacgatcatcggtgtgcccggcagggtgaaaggtctgtccggcggagaaaggaagcgtctggcattcgcctccgaggcactaaccgatccgccgcttctgatctgcgatgagcccacctccggactggactcatttaccgcccacagcgtcgtccaggtgctgaagaagctgtcgcagaagggcaagaccgtcatcctgaccattcatcagccgtcttccgagctgtttgagctctttgacaagatccttctgatggccgagggcagggtagctttcttgggcactcccagcgaagccgtcgacttcttttcctagtgagttcgatgtgtttattaagggtatctagcattacattacatctcaactcctatccagcgtgggtgcccagtgtcctaccaactacaatccggcggacttttacgtacaggtgttggccgttgtgcccggacgggagatcgagtcccgtgatcggatcgccaagatatggcgacaattttgctattagcaaagtagcccgggatatggagcagttgttggccaccaaaaatttggagaagccactggagcagccggagaatgggtacacctacaaggccacctggttcatgcagttccgggcggtcctgtggcgatcctggctgtcggtgctcaaggaaccactcctcgtaaaagtgcgacttattcagacaacggtgagtggttccagtggaaacaaatgatataacgcttacaattcttggaaacaaattcgctagattttagttagaattgcctgattccacacccttcttagtttttttcaatgagatgtatagtttatagttttgcagaaaataaataaatttcatttaactcgcgaacatgttgaagatatgaatattaatgagatgcgagtaacattttaatttgcagatggttgccatcttgattggcctcatctttttgggccaacaactcacgcaagtgggcgtgatgaatatcaacggagccatcttcctcttcctgaccaacatgacctttcaaaacgtctttgccacgataaatgtaagtcttgtttagaatacatttgcatattaataatttactaactttctaatgaatcgattcgatttaggtgttcacctcagagctgccagtttttatgagggaggcccgaagtcgactttatcgctgtgacacatactttctgggcaaaacgattgccgaattaccgctttttctcacagtgccactggtcttcacggcgattgcctatccgatgatcggactgcgggccggagtgctgcacttcttcaactgcctggcgctggtcactctggtggccaatgtgtcaacgtccttcggatatctaatatcctgcgccagctcctcgacctcgatggcgctgtctgtgggtccgccggttatcataccattcctgctctttggcggcttcttcttgaactcgggctcggtgccagtatacctcaaatggttgtcgtacctctcatggttccgttacgccaacgagggtctgctgattaaccaatgggcggacgtggagccgggcgaaattagctgcacatcgtcgaacaccacgtgccccagttcgggcaaggtcatcctggagacgcttaacttctccgccgccgatctgccgctggactacgtgggtctggccattctcatcgtgagcttccgggtgctcgcatatctggctctaagacttcgggcccgacgcaaggagtagccgacatatatccgaaataactgcttgtttttttttttaccattattaccatcgtgtttactgtttattgccccctcaaaaagctaatgtaattatatttgtgccaataaaaacaagatatgacctatag

**E=Eye enhancer (inserted in reverse orientation relative to 5’construct end):**

acccagaccaacccccccaaccccccaccagaccgcacttcctgtgacaatggctggggacttacctttgccgatatatatatatatatatatatatatacatatgtatgtaggtgtgactgtgcggagagagtataaaagtgtgtgttgaaaaaaactttctacgcctcagttcaagttacttccttttcgcttttcaatacggtgttattgcattgcattgtggaatagcaaaccaatttaaagaatcccaaaggtagggcaaaaaaaaatgttcaattgcagggctaataagcaaaaatcaagttaaggccactctaaaaaaaatgaactgcatacttatacgtatgcaattaactttattattgatttagagcgcataaaaacggcaaactaattaattacagcattcccttagagcatttcataatcggttttaagcactttttgttgtaatgccatttttcagctctttcgctgctgcgacaggcgagtgacaataaattgttaagtgcaaaaagtttacagcatttgagtgagttagtaatggcaaacacacacgaacacgaacttcaagacggtctaaacccgattcgagctccactcaaccaacatcgaagaagccccataagtgtcactgagcgaaaaactcggttagttggcaaaaat

**(2)**

**Details of *Drosophila* genetic crosses**

1. The following steps were made to obtain homozygote transgenes lines:
2. For the transgenes on the second chromosome (inserted into ZH-attP-22A, ZH-attP-51C or ZH-attP-58A) the yacw1118 balancer stock carrying dominant marker In(2RL),CyO was used. Flies with the second chromosome balancer, CyO, have curly wings.

F0: ♂ *yacw^1118^; K//+;+* r *♀* *yacw^1118^*; *CyO//+;+*

*↓*

F1: ♂ *yacw^1118^; K//CyO; +* r *♀ yacw^1118^; K//CyO; +*

*↓*

F2: ♂ *yacw^1118^; K//K; +* r*♀* *yacw^1118^; K//K; +*

*↓*

*stock*

*(Note:* ♂ and *♀yacw^1118^; CyO//CyO; + are lethal)*

1. For the transgenes on the third chromosome (inserted into ZH-attP-68E, ZH-attP-96E) the yacw1118 balancer stock carrying dominant marker In(3LR)TM3,Sb was used. Flies with the third chromosome balancer, TM3,Sb have Stubble (Sb) bristles.

F0: ♂ *yacw^1118^; +; K//+* r *♀* *yacw^1118^*; *+;TM3,Sb//+*

*↓*

F1: ♂ *yacw^1118^; +; K//TM3,Sb* r *♀ yacw^1118^; +; K//TM3,Sb*

*↓*

F2: ♂ *yacw^1118^; +;K//K* r*♀* *yacw^1118^; +; K//K*

*↓*

*stock*

*(Note:* ♂ and *♀yacw^1118^; TM3,Sb//TM3,Sb; + are lethal)*

1. For the analysis to obtain hemizygotes homozygote stocks were crossed with *yacw^1118.^* flies.
2. Trans-heterozygotes were obtained by crossing homozygotes of corresponding lines.
